# Prediction of functionally important residues in globular proteins from unusual central distances of amino acids

**DOI:** 10.1186/1472-6807-11-34

**Published:** 2011-09-18

**Authors:** Marek Kochańczyk

**Affiliations:** 1 Faculty of Physics, Astronomy and Applied Computer Science, Jagiellonian University, ul. Reymonta 4, 30-059 Krakow, Poland; 2 Institute of Fundamental Technological Research, Polish Academy of Sciences, ul. Pawińskiego 5B, 02-106 Warsaw, Poland

## Abstract

**Background:**

Well-performing automated protein function recognition approaches usually comprise several complementary techniques. Beside constructing better consensus, their predictive power can be improved by either adding or refining independent modules that explore orthogonal features of proteins. In this work, we demonstrated how the exploration of global atomic distributions can be used to indicate functionally important residues.

**Results:**

Using a set of carefully selected globular proteins, we parametrized continuous probability density functions describing preferred central distances of individual protein atoms. Relative preferred burials were estimated using mixture models of radial density functions dependent on the amino acid composition of a protein under consideration. The unexpectedness of extraordinary locations of atoms was evaluated in the information-theoretic manner and used directly for the identification of key amino acids. In the validation study, we tested capabilities of a tool built upon our approach, called SurpResi, by searching for binding sites interacting with ligands. The tool indicated multiple candidate sites achieving success rates comparable to several geometric methods. We also showed that the unexpectedness is a property of regions involved in protein-protein interactions, and thus can be used for the ranking of protein docking predictions. The computational approach implemented in this work is freely available via a Web interface at http://www.bioinformatics.org/surpresi.

**Conclusions:**

Probabilistic analysis of atomic central distances in globular proteins is capable of capturing distinct orientational preferences of amino acids as resulting from different sizes, charges and hydrophobic characters of their side chains. When idealized spatial preferences can be inferred from the sole amino acid composition of a protein, residues located in hydrophobically unfavorable environments can be easily detected. Such residues turn out to be often directly involved in binding ligands or interfacing with other proteins.

## Background

The task of assigning a function to each new protein structure resulting from high-throughput structural genomics experiments requires reliable computational annotation methods. Identified functionally important amino acids can provide preliminary clues on the co-evolution and molecular workings of proteins. Such information is crucial for the site-directed mutational engineering and *de novo *protein design. The integration of knowledge of the locations of binding sites with ligand screening or docking protocols improves initial stages of the rational drug design [1]. Also, when putative residues responsible for the complex formation are identified, protein-protein interaction interfaces can be characterized *in silico *[2].

Currently, due to the availability of 3D data, the exploration of properties embedded in the structure of proteins prevails over the traditional motif recognition and sequence comparison (that may turn out to be surprisingly ambiguous [3]). For close homologs, the knowledge-based approaches transfer functional annotations from proteins with already known structure and function [4-8]. Their average effectiveness is inherently limited by the availability of solved and annotated structures, so more generic methods are still desirable. Numerous pure geometry-based methods search locally for clefts and pockets in the molecular surface by employing computational geometry algorithms [9-16]. The spatial neighborhood of residues is used to characterize local environments in methods that take into account additional factors such as the flexibility of residues [17], electrostatic potential [18,19] or overall interaction energy [20], excess or deficiency of the hydrophobicity [21], hydrophobic potential around a protein [22] or a multitude of other, predominantly physicochemical, residue properties [23-27].

Interestingly, indications based on diverse descriptions are usually not correlated [28]; nor can they be used for the prediction of both protein-ligand and protein-protein interaction sites [29]. As a consequence, well-performing present-day approaches use combinations of complementary characteristics, for example the electrostatics and geometric properties [30] or the geometry and conservation [31-33]. Metaservers offer combinations of several independent fully-fledged methods in order to compensate for the shortcomings of some methods with capabilities of others [34,35]. As the compositions of distinct binding site prediction methods achieve better success rates than constituent techniques applied solo, it is still valuable not only to provide fine-tuned variations of heterogeneous approaches, but also to search for assorted methods that could complement existing ones by the exploration of specific orthogonal features.

Contrary to the majority of approaches that characterize fragments of proteins locally and with a considerable degree of detail, Brylinski et al. [21,36] showed that the rough analysis of the global spatial distribution of amino acids with respect to their hydrophobicity is capable of localizing ligation sites. They did not follow usual hydrophobicity quantifications such as the average solvent-accessible surface area or number of contacts [37], but rather measured the discrepancy between idealized and observed hydrophobicity within the fuzzy oil drop model [38], where the trivariate Gaussian distribution is used to express the idealized protein hydrophobicity (maximum value in the protein core, smoothly approaching 0 about and beyond the perimeter). It turned out that amino acids of high discrepancy (unexpectedly high hydrophobicity in relation to their peripheral position) often occur in function-related areas of proteins.

This observation is fundamental to the current work, where we devised and validated a method for the identification of function-related residues based on the probabilistic description of atomic burials originating from the conceptual framework of Gomes et al. [39]. We collected necessary statistics from a selection of globular proteins and, as opposed to the original application of the framework, we used a radial probability density function to describe preferred central distances of individual atoms of types defined within amino acids. In this view, proteins are treated as mixtures of amino acids where restraints resulting from their covalent connectivity are ignored (except for cysteines). Any deviations from the spherical shape of the macromolecule, intrinsic rigidness imposed by the presence of secondary structures and local interactions are neglected: proteins are treated as compact solid-like bodies of atoms, where the isotropic hydrophobic segregation and packing are considered to be the dominant driving forces conferring spatial organization of residues [40-42].

The classic analysis of just several protein structures suggested that the sole orientational preferences of side chains can be a criterion for the hydrophobic or hydrophilic character [43]. Therefore, although a multitude of hydrophobicity scales or burial indices are available for (whole) amino acids and many knowledge-based pair-potentials are constructed for (united) residue side chains [44], we decided to act on the per-atom rather than per-residue basis in order to account for (radial) orientational preferences of residues. The actual amino acid composition of a protein influences its native structure topology [45,46], folding type [47,48] and interactions [49]. In our statistical model, for a protein with a known amino acid abundance we assume that the relative probabilities are directly proportional to the stoichiometry. In our approach to the function prediction, every heavy atom in every amino acid of the protein considered has the measure of its *unexpectedness *estimated with respect to all possible atom types in a given point of space. The measure depends solely on the distance from the geometric center of the polymer. Typically, residues that place their atoms in the least probable central distances appear to contribute to the creation of ligand binding sites (including active sites of enzymes) or protein-protein binding interfaces.

## Methods

### Extraction of a non-redundant set of globular proteins

We examined a total of 172 265 protein chains as deposited in RCSB PDB [50] in January 2011 and excluded structures of high asymmetry or in other aspects irregular. Two geometric descriptors were used discriminatively: asphericity, calculated as the normalized sum of squared differences of the eigenvalues of the gyration tensor (according to [51]), was required to be smaller than 0.1 and compactness to be at least 0.5; the latter value was calculated as the ratio of the solvent accessible surface area of the (ideal) sphere of the volume of a considered protein to its actual solvent accessible surface area (this is a more intuitive inverse of the fraction introduced by Galzitskaya et al. [52]). Chains of sequence lengths smaller than 100 amino acids were excluded due to strong geometric constraints. Proteins that fulfill all the aforementioned conditions are denoted as globular in this paper.

Furthermore, it was required that every solved structure should contain no discontinuities, be determined with an experimental method to a resolution better than 2 Å, contain only a single domain (according to both SCOP [53] and CATH [54] classifications) and must not create multi-chain complexes, even transiently (determined on the basis of biological units assemblies available from PDB). A total of 2953 proteins were extracted for further considerations (1.71% of the whole PDB).

In the last step, in order to reduce sequence redundancy, precomputed clustering results available from the PDB, generated by the Cd-hit program [55] that grouped sequences of at least 90% of sequence identity in clusters, were used to select a single protein per every cluster. Finally, the learning data set comprised 775 high-resolution single-domain globular chains (26.2% of previously selected chains). The full list of PDB ids is available in Additional file 1 Table S1.

Compactness and asphericity of proteins in the set turned out to be only weakly interdependent (correlation coefficient, CC, -0.14). Longer chains were characterized by lower compactness (CC = -0.45) but not necessarily higher asphericity (CC = -0.06). Distributions and dependencies of geometric descriptors are presented in the Additional file 2 Figure S1.

### Probabilistic description of atomic burials

Geometric centers and radii of gyration were calculated for every chain in the learning set. Distances to the geometric center of a chain of every heavy atom, *r*, were divided by the radius of gyration of the whole chain, *r_g_*, enabling a uniform view of globular proteins of various sizes [43]. Histograms of such normalized distances, *R *= *r/r_g_*, were collected for every amino acid-dependent atom type denoted by *τ*. Three types of cysteines were considered separately: generic Cys (irrespective of the presence or absence of SS bonding), Cys creating (intra-chain) disulfide bridges (denoted CSS, nearly 40% of all Cys) and Cys reduced and not involved in SS bridging (C_SH_). A total of 170 histograms for different *τ *were obtained.

A continuous "mass" function derived by Gomes et al. [39] to describe burials of whole residues was considered for fitting. The original function expresses the quadratic increase of the volume when moving away from the core of a protein and sigmoidal decrease (Fermi function) of the atomic density in the rim as dependent on the normalized radius, *R*:

(1) $$p_{\alpha }\left(R;\tau \right)=\frac{A_{\tau }\;R^{2}}{1+\exp\left(\beta_{\tau }\left(R^{\alpha_{\tau }}-\mu_{\tau }\right)\right)}\text{.}$$

After applying the direct least-squares method for fitting individual histograms, obtained fits yielded unsatisfactory sums of the squared residuals (SSR) for atoms in hydrophilic residues, where the expression overestimated their propensity to occur in the protein core. To account for this observation, the assumption of the strictly quadratic increase was abandoned and an additional tunable parameter, *γ_τ_*, was introduced while *α_τ _*was set to 1 (see Additional file 3 Figure S2). The following form was finally used:

(2) $$p\left(R;\tau \right)=\frac{A_{\tau }R^{\gamma_{\tau }}}{1+\exp\left(\beta_{\tau }\left(R-\mu_{\tau }\right)\right)}$$

for fitting. Parameter *A_τ _*provides normalization, *μ_τ _*principally determines location, *β_τ _*influences the width of the distribution and *γ_τ _*controls convexity of the left ridge. The goodness-of-fit of distributions of the latter form was better for 124 of 170 fits (in terms of SSR) in comparison to the original distribution function with variable *α *(Equation 1) and for 130 of 170 fits (F-test with *p*-value < 0.000001) in comparison to the original distribution function with *α *= 1.

### Expected atomic burials in proteins

Densities of atoms are characterized globally in the environment of the protein itself in the common and reduced coordinate space. Thus, assuming the lack of void spaces inside, in a given point in space, located in the normalized distance *R *from the geometric center of the protein, one can estimate the expected chance of occurrence of an atom *τ *by relating its probability, *p*(*R*; *τ*), to probabilities of occurrences of all atoms, Σ_*τ*∈*T *_*p*(*R*;*τ*), where *T *is the complete set of 170 atomic types. As we consider concrete protein species, probabilities depend effectively on the number of atoms *τ *(equal to the number of amino acids of a concrete type) present in the whole protein, *n*(*τ*). Only their relative fractions are important so we can use them directly for weighting in the expression similar to the posterior distribution of component membership in mixture models. The equation

(3) $$\bar{p}\left(R;\tau \right)=\frac{n\left(\tau \right)p\left(R;\tau \right)}{\sum_{\tau^{\prime }\in T}n\left(\tau^{\prime }\right)p\left(R;\tau^{\prime }\right)}$$

is used for the estimation of expected atomic central distances in proteins with known amino acid composition. The variability of preferred atoms in a given point in space is measured in bits as the entropy of expected burials:

(4) $$S\left(R\right)=-\underset{\tau \in T}{\sum }\bar{p}\left(R;\tau \right)\log_{2}\bar{p}\left(R;\tau \right).$$

### Prediction of functionally important residues

In search of residues employed directly in performing the function, we follow the crucial observation by Brylinski et al. [56] that irregularities in the global distribution of hydrophobicity often indicate function-related areas. We follow this principle in our probabilistic approach by searching for atoms of the relatively least probable central distances, $\bar{p}\left(R;\tau \right)$. Residues with such atoms are usually the hydrophobic amino acids exposed to the solvent or hydrophilic amino acids located close to the protein core. The unexpectedness of a central distance can be converted into a simple free energy-like term by the following equation:

(5) $$Unexpectedness\left(R;\tau \right)=-\log_{2}\bar{p}\left(R;\tau \right),$$

which gives estimates in bits.

#### Prediction of ligand binding sites

As for compact structures it holds that *r_g _*is roughly proportional to (sequence length)^1/3 ^[57] and as in the task of binding sites recognition one is interested primarily in non-buried residues on the surface, the area of which is proportional to $r_{g}^{2}$, as a rule of thumb, $\left\lfloor \frac{1}{4}\cdot {\left(\text{sequence length}\right)}^{2∕3}\right\rfloor$ residues containing the most unexpected atoms are initially selected. (However, assuming the general spatial character of the statistical model, no additional factors such as estimates of solvent accessibility are taken into account.) Selected residues are weighted proportionally to the maximum value of unexpectedness among values assigned to constituent atoms and then clustered hierarchically using the pairwise average-linkage method. In search for ligand binding sites, the hierarchy of residues is partitioned into clusters separated by more than 7 Å (average Euclidean distance) that indicate (possibly multiple) putative sites. Positions of cluster centroids are computed in a weighted manner and located closer to the most unexpected atoms. Putative sites are ranked according to the proximity of their predicted centroids to the geometric center of the whole protein.

#### Prediction of protein-protein interfaces

Contrary to the development of the complete algorithm for the prediction of binding sites of (small) ligands, we do not attempt to create a new protein-protein docking method but rather to provide a simple unexpectedness-based scoring function for the ranking of docking predictions. Heavy atoms of one protein located within a distance of 10 Å from the other have their unexpectedness calculated and a maximum value of unexpectedness is found in this way for both macromolecules of a docked assembly. A docking prediction is then scored using the average of the highest values of unexpectedness in two interfaces.

### Evaluation of predictions

The evaluation of the method based on the introduced characteristics was performed separately for the task of predicting binding sites of small ligands and for the prediction of regions creating interfaces to other proteins. In both cases, if a test data set allowed, predictions were made for unbound structures; after the assignment, the *apo *form was superimposed onto the *holo *form so that intermolecular distances were measured between the unbound structure and ligand/another macromolecule as located in the structure of the complex.

For the prediction of ligand binding sites, a set of 48 pairs of unbound/bound structures and a set of 210 bound structures, which were already employed for the benchmarking of other methods (LigSite^csc ^[32] and IBIS [8]), were used for the comparison with already measured success rates of the state of the art geometry-based methods: SURFNET [9], PASS [10] and LigSite [12]. The former set, further referred to as the LB_48_ test set, includes 38 enzymes that cover 39 diverse enzymatic activities according to the EC annotations from the Catalytic Sites Atlas version 2.2.12 [58] and 10 proteins that bind compounds in their non-active sites. The latter set, referred to as the LB_210 _test set, enabled large-scale benchmarking.

In order to juxtapose the results of our approach and similar fuzzy oil drop-based method (FOD), which assign prediction scores to clusters of atoms, with pocket identification methods, which indicate geometric centers of pockets located over the molecular surface, we used MSMS [59] and projected coordinates of centroids of putative binding sites onto the solvent-excluded molecular surface. Then, in order to apply the cut-off value of 4 Å used in pocket prediction benchmarks, we displaced surface-projected coordinates by 1 Å in the direction of the vector normal to the surface and 1 Å outwards from the geometric center of the protein. As the points do not always lie the space in the pocket, additionally we used the cut-off of 6 Å. We examined whether any atom of the ligand is located within the cut-off distance and reported success rates for the best ranked (Top 1) and 3 highest ranked (Top 3) candidate sites.

In order to show, preliminarily, that the unexpectedness is a property of protein-protein interfaces, we used the latest and most extensive docking benchmark (version 4.0) [60], further referred to as the PPI_176 _test set. Residues of two macromolecules were considered as interfacing if they were separated by at most 4 Å. In the case of protein-protein binding interfaces, unexpected residues are usually isolated, so we did not cluster them, but rather reported the average unexpectedness in binding/non-binding protein regions.

Eventually, the capability of appropriate ranking of protein-protein docking predictions was compared to that of one of the best performing docking algorithms, ZDock [61], optionally amended with ZRank [62], and two other methods, recent ASP-Dock [63] and older FTDock [64]. The methods have their success rates already measured over the complete protein docking benchmark version 3.0 [65], so this set (referred to as the PPI_124 _test set) was used to estimate the capacity of our approach. The unexpectedness-based score assessed 54,000 docking poses of a decoy generated by ZDock 3.0 operating at the rotational scanning interval of 6°. A successful prediction was defined as a docking solution of ligand C*^α ^*RMSD < 10 Å.

### Comparison with other characteristics

A direct evaluation of the current method was performed in parallel with the fuzzy oil drop (FOD) method [21] using the LB_48 _test set. The same clustering and ranking methods were used for residues with the highest unexpectedness and for residues of the highest observed vs. theoretical hydrophobicity discrepancy, $\Delta \tilde{H}$ (FOD). For the detailed comparison with other explorable characteristics, useful for the prediction of (small) ligand binding sites, the evolutionary conservation scores were assigned to residues according to the multiple-sequence alignment-based ConSurf-DB [66]; only residues of the highest conservation score (i.e. 9) are indicated in this paper. Independently, the clusters of ionisable residues with anomalous predicted titration behaviour, identified with the finite difference Poisson-Boltzmann-based technique, Thematics [25], were included in the comparison.

## Results

### Orientational preferences of amino acids

Parameters of probability distribution functions given by Equation 2, *A_τ _*, *μ_τ _β_τ _*and *γ_τ_*, were determined independently for every amino acid-dependent atom type, *τ*, allowing to capture the specific radial orientational propensities of amino acids. The full list of 170 sets of parameters for atomic distribution functions derived from the obtained learning set can be found in the Additional file 4 Table S2. Since the structure of side chains allows to single out the atom most distant from the C*^α ^*atom, it is possible to capture and demonstrate preferred orientations using a less redundant description. We decided to evaluate unexpectedness of every atom uniformly motivated by the fact that among 83 distributions of all side chain heavy atom types as many as 58 were statistically significantly different than distributions of relevant C*^α ^*atoms (Kolmogorov-Smirnov tests with *p*-value < 0.000001; see Additional file 4 Table S2 for details).

**Figure 1:**
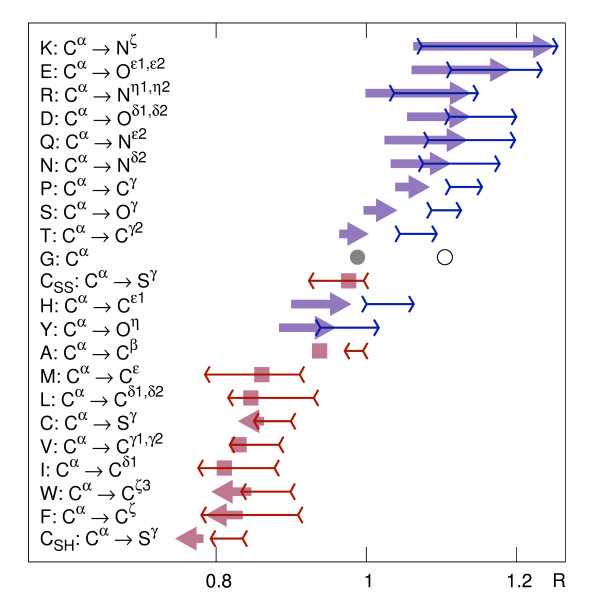
**Orientational preferences of amino acids in globular proteins**. Locations of mean and maximum values of probability density functions for C*^α ^*and most distant side chain atoms for all amino acids. Thick arrows connect means; thin arrows span between maxima of distributions. All arrows point towards the most distal atom in the side chain (except for Gly) according to the labels on the left. The arrows that would be shorter than their heads are replaced by squares.

**Figure 2:**
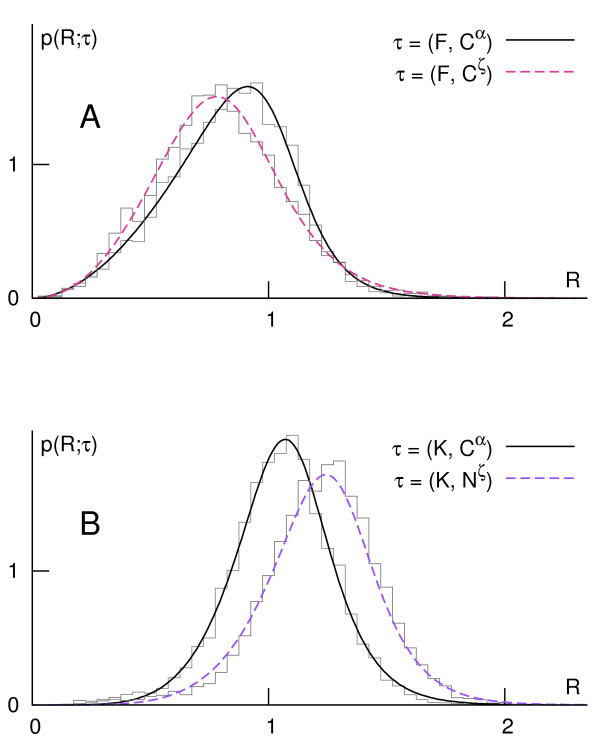
**Probability densities of C***^α ^***and of the most distal side chain atom in Phe and Lys**. The two amino acids exhibit the most prominent (the largest separation of the means) and opposite (centripetal, **A**, vs. centrifugal, **B**) orientational preferences.

Resulting probability density functions have nonzero skewness, so in order to portray synthetically the orientational preferences, we use both differences between mean values and between maxima of distributions of C*^α ^*and distal atoms (Figure 1). The arrows can be interpreted as expressing global hydrophobic moments of (amphiphilic) residues defined in the environment of the protein itself (analogous to [67]). In this view, the two amino acids of the most prominent opposite orientational preferences are Lys and Phe (Figure 2).

Although side chains determine the hydrophobic/hydrophilic character of amino acids, they influence considerably probabilities of spatial occurrence of (chemically equivalent across amino acid types) C*^α ^*atoms. In the synthetic picture of atomic densities (Figure 1 and Additional file 5 Figure S3), hydrophobic propensities of amino acids in the body of a protein are modulated by their sizes: broad distributions of Gly and Ala atoms are shifted from those of other hydrophobic types; distributions of large amino acids, such as Trp or Arg, are less dispersed around their maxima; the broad distribution of His can be explained by diverse possible protonation states and the ambivalent distribution of Tyr - by mixed aromatic/polar character of its side chain.

The analysis of the intriguing case of Cys reveals that, although their orientation does not depend on the possible disulfide bonding, the non-bridging cysteines prevail as the most buried residues, while those constituting cystines occur more often on the protein surface (Figure 1; Additional file 6 Figure S4). Cysteines are relatively frequently found in active sites [68]; supposedly, the evolution may easily redefine the function of a protein by tailoring the state of cysteines and adjusting their positions [69].

### Distribution of unexpectedness

The mean central reduced distances of distal site chain atoms are in agreement with known hydrophobicity scales, especially those empirical ones based on the surface accessibility. Several theoretical and one experimental scale, along with similarities expressed in terms of the correlation coefficient, are listed in Table 1.

**Table 1:** Correlations of mean values of distal side chain atom distributions to other characteristics

CC	Description of the characteristics	Reference
-0.984	Mean fractional area loss upon folding	[88]
-0.974	Solvent accessibility based on self-information [16% accessibility]	[89]
-0.971	Information value for accessibility [average fraction 35%]	[90]
+0.961	Normalized eigenvector of the Sweet & Eisenberg scale	[91]
-0.951	Mean combined polarity calculated from distributions of residues in proteins	[92]
+0.897	Hydrophobicity coefficient in RP-HPLC [C4 with 0.1%TFA/MeCN/H_2_O]	[93]

Similarities of 5 theoretical (top) and 1 experimental (bottom) single-value amino acid characteristics are expressed in terms of the correlation coefficient, CC. (For Cys, the distribution of reduced S*^γ ^*was used.)

**Figure 3:**
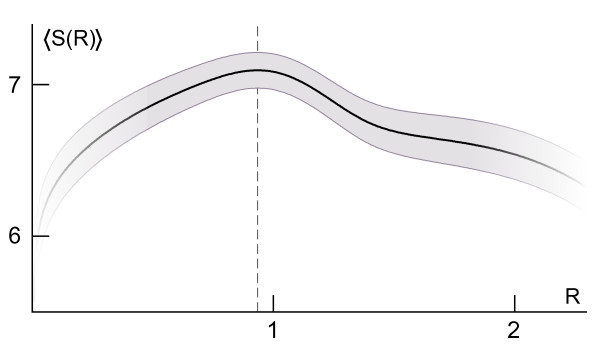
**Entropy of expected reduced central distances in globular proteins**. The entropy, *S*(*R*), of $\bar{p}\left(R;\tau \right)$ was averaged over all proteins from the learning set and is expressed in bits (black line; gray band - standard deviation of the mean entropy; dashed line - location of the maximum). "Twilight zones" mark regions where the entropy was calculated using tails of distributions.

The statistical model applied to globular proteins from the learning set reveals a critical value of about 0.93 · *r_g_*, where the average entropy, calculated according to the Equation 4 and interpreted as the lack of preference for particular atomic types, has the highest value (Figure 3). The value marks clearly the hydrophobic-hydrophilic transition on the protein surface, usually covered by a patchwork of hydrophobic and hydrophilic areas [70,71]. Although it was observed in larger proteins that the degree of hydrophobicity is constant for *R <*0.7 [72], according to the model the protein interior is not a volume of uniform preferences, but rather it visibly exhibits a gradually increasing preference for some apolar atomic types (decreasing entropy) when moving towards the centroid.

**Figure 4:**
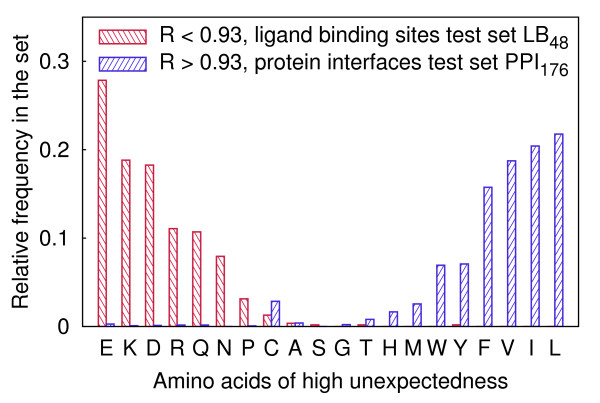
**Relative frequencies of amino acids characterized by high unexpectedness**. Residues lying within 0.93 · *r_g_* in proteins from a set used for the ligand binding site prediction and residues of central distances greater than 0.93 · *r_g _*from a set used for the protein-protein interface prediction are presented separately.

Types of the most unexpected amino acids (i.e. amino acids comprising most unexpected atoms) were determined in the LB_48 _test set and in the PPI_176 _test set separately (Figure 4). In the former set, the additional requirement of *R <*0.93 and in the latter the requirement of *R >*0.93 were imposed, because several proteins in the LB_48 _test set create complexes with other proteins and proteins in the PPI_176 _test set contain ligand binding pockets. According to the model, the most unexpected residues lying within the radius of gyration are those charged or ionizable, such as Glu, Asp, Lys and Arg, which are known to play essential functional roles in the enzymatic active sites. Amino acids with branching aliphatic side chains, Leu, Val and Ile, are properly assessed as being rarely exposed to the solvent. Unfortunately, broad distributions of central distances of His and Tyr cause them to be hardly ever indicated as unexpected. Also, due to the specific structural roles of Pro and Cys, such residues tend to be rated as unexpected despite the possible lack of any direct relation to the function.

### Prediction of ligand binding sites

Clusters of unexpected residues turn out to be located on the surface of proteins, very often inside clefts and pockets, where ligand compounds are bound. Geometric centroids of such clusters designate candidate ligand binding sites with the success rate similar to that of the fuzzy oil drop-based method in the LB_48 _test set and only slightly worse in the LB_210 _test set (see Table 2). For the cut-off value of 6 Å of the distance to a ligand, considered as enabling the comparison, the performance of both global hydrophobicity distribution-based strategies is similar or even marginally better than that of three state of the art methods, PASS, LIGSITE and SURFNET, which distinguish clefts or cavities based solely on the local geometry (Table 2).

**Table 2:** Benchmarks of several ligand binding site prediction methods

	LB_48 _test set		LB_210 _test set	
		
Method	Top 1	Top 3	Top 1	Top 3
PASS	60*	71*	54*	79*
LIGSITE	58*	75*	65*	85*
SURFNET	52*	75*	42*	56*
FOD	56 (71)	60 (81)	55 (68)	72 (83)
Unexpectedness	48 (69)	63 (83)	53 (65)	67 (80)

The comparison of ligand binding site prediction success rates of the current approach (Unexpectedness), a global hydrophobicity-based method (FOD) and several non-hybrid pocket-searching state of the art methods for 48 unbound molecules from the LB_48 _test set. The cut-off distances are 4 Å and 6 Å (success rates for the latter value are in parentheses). Results marked with stars were reported in [32].

The relations to other characteristics frequently exploited for the localization of binding sites, viz., conservation and electrostatics, were examined for residues in properly indicated Top 3 clusters (Table 3). There are no clusters with active site residues displaying neither conservation nor the indicative anomalous ionisable behavior - in fact, in most cases there is a significant overlap between the unexpectedness and two other attributes; in remaining cases the three features may be seen as complementing one another (especially for residues that are nonionizable or bind with low specificity).

**Table 3:** Residues in correctly predicted 3 top-ranked clusters

Structure	Function	Cluster
1ahc A	(RNA) glycosidase	$\underline{\text{R}},\underline{\ddot{\text{E}}},\ddot{\text{E}},\underline{\text{Q}}$
1bbs A	proteinase	$\underline{\ddot{\text{D}}},\underline{\ddot{\text{D}}}$
1bya A	O-glycosidase	$\underline{\ddot{\text{E}}},\underline{\text{R}},\underline{\ddot{\text{E}}},\text{P}$
1cge A	metalloproteinase	$\underline{\ddot{\text{E}}}$
1djb A	hydrolase (*β*-lactamase)	$\underline{\text{K}},\underline{\text{E}}$
1hsi A	protease (HIV-2 retropepsin)	$\underline{\text{I}}\;\left(\text{flaps}\right)$
1hxf H	(serine) protease	$\underline{\ddot{\text{D}}}$
1ifb A	*fatty acid binding*	$\ddot{\text{R}},\text{E}$
1ime A	(inositol) phosphatase	$\underline{\ddot{\text{D}}},\underline{\ddot{\text{D}}},\underline{\ddot{\text{D}}}$
1krn A	hydrolase (fibrinolysin)	$\ddot{\text{K}}$
1l3f E	proteolysin	$\underline{\text{E}}$
1nna A	O-glycosidase	$\underline{\ddot{\text{K}}},\underline{\ddot{\text{E}}},\underline{\text{R}},\underline{\ddot{\text{E}}},\underline{\text{Q}}$
1npc A	(metallo)protease	$\underline{\text{E}},\underline{\text{E}}$
1pdy A	enolase	$\underline{\text{K}},\underline{\text{R}},\underline{\text{Q}}$
1psn A	(acid) proteinase	$\underline{\ddot{\text{D}}},\underline{\ddot{\text{D}}}$
1pts A	*azobenzoic acid binding*	$\underline{\text{D}}$
1qif A	(acetylcholin)esterase	$\underline{\ddot{\text{E}}}$
1stn A	(phosphodi)esterase	$\underline{\text{R}},\underline{\ddot{\text{D}}}$
1ypi A	(triosephosphate) isomerase	$\underline{\text{K}},\underline{\text{E}}$
2cba A	lyase (anhydrase)	$\underline{\ddot{\text{E}}},\underline{\ddot{\text{E}}}$
2ctb A	hydrolase (carboxypeptidase)	$\underline{\text{E}}$
2fbp B	(fructose bis)phosphatase	$\underline{\text{K}},\underline{\ddot{\text{E}}},\underline{\text{D}},\underline{\ddot{\text{D}}},\underline{\ddot{\text{E}}}$
2sil A	hydrolase (neuraminidase)	$\ddot{\text{E}},\text{Q},\text{Q},\underline{\ddot{\text{R}}},\underline{\ddot{\text{R}}}$
3app A	(acid) proteinase	$\underline{\ddot{\text{D}}}$
3p2p A	(carboxyl)esterase	$\text{R},\underline{\text{D}}$
3ptn A	hydrolase (tripsin)	$\underline{\text{D}}$
3tms A	(methyl)transferase	$\underline{\ddot{\text{E}}},\underline{\text{N}},\underline{\text{Q}}$
5dfr A	(folic acid) reductase	$\underline{\text{D}}$
8adh A	dehydrogenase	$\underline{\text{E}},\underline{\ddot{\text{D}}}$
8rat A	hydrolase (ribonuclease)	$\underline{\ddot{\text{K}}},\text{Q}$

Residues are sorted in rows according to decreasing unexpectedness. Residues of the highest evolutionary conservation scores according to the ConSurf-DB [66] are underlined; residues indicated as functional by Thematics [25] have overbars; bold residues are annotated as catalytic in the Catalytic Sites Atlas (CSA) [58]. (Two chains of non-enzymatic functions are unannotated in the CSA.)

Among the proteins annotated with EC numbers in the LB_48 _test set, 35 out of 38 enzymes have their active sites recognized in Top 3 clusters (31/38 in Top 1). Notwithstanding, out of 10 proteins that exhibit no enzymatic activity and bind ligands in their non-active sites, binding sites are properly recognized in only 5 cases, mainly because of their eccentric locations (see Additional file 7 Table S3 for details).

**Figure 5:**
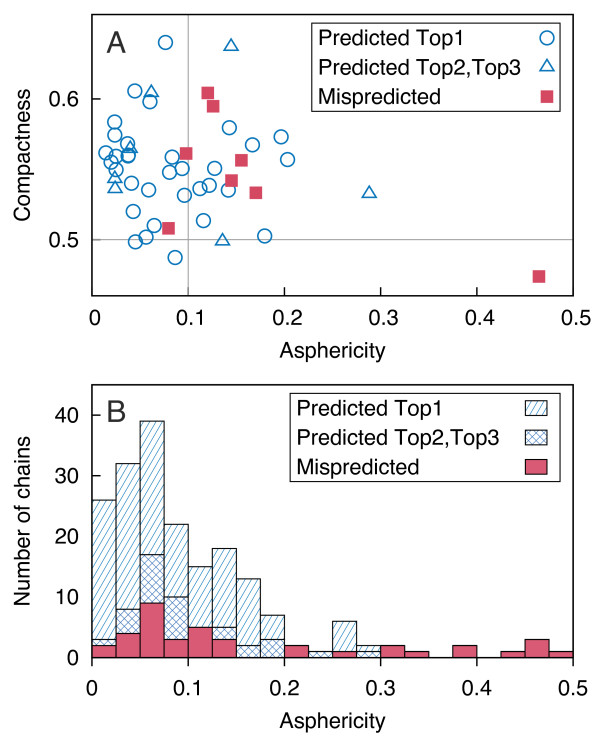
**Dependence of the prediction success on geometric characteristics**. Predictions for apoproteins from the LB_48 _test set (**A**) and holoproteins from the LB_210 _test set (**B**) are shown (cut-off 6 Å). Two perpendicular thin gray lines correspond to the geometric requirements imposed on proteins in the learning set.

The predictive power of our approach decreases moderately for more aspherical proteins. The quality of cluster rankings seems to be independent of the asphericity (Figure 5).

### Ranking of protein-protein docking results

**Figure 6:**
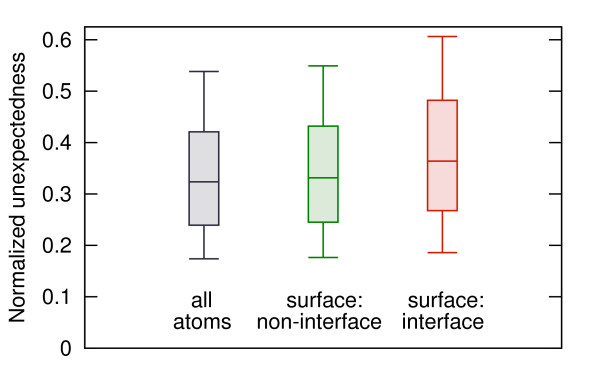
**Unexpectedness of atoms in protein interfaces**. Normalized values of unexpectedness for all atoms and atoms belonging to residues exposed to the solvent (residue solvent-accessible area *>*10 Å^2^), subdivided into these creating and not creating protein-protein interfaces. Whiskers represent the 9^th ^and the 91^st ^percentile.

The unexpectedness was employed to characterize the protein-protein interfaces in the PPI_176 _test set, where the majority of structures have the asphericity higher than 0.1. Despite this difficulty, the median unexpectedness of interacting residues turns out to be clearly higher than the median unexpectedness of all surfaces residues (Figure 6). When a subset of more globular proteins is examined, the difference is even more salient (not shown).

**Figure 7:**
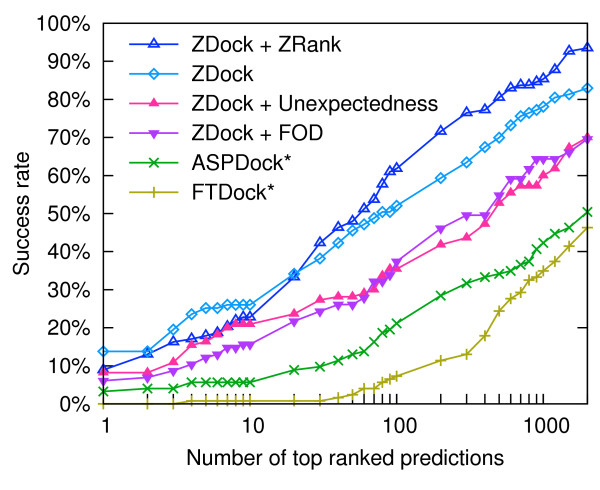
**Effectiveness of rankings of docking solutions**. Decoys generated and ranked by ZDock were reranked using ZRank, $\Delta \tilde{H}$ (FOD) and the unexpectedness. Success rates of two independent docking approaches, FTDock and ASPDock, marked with stars, are displayed as reported in [63].

Scoring of interfaces based on the unexpectedness yields consistently better results than an analogous FOD-based scoring for 100 top-ranked solutions (Figure 7). For 10 top-ranked docking solutions success rates of our approach are nearly comparable to that of the ZRank, indicating that our score can properly account for desolvation and electrostatics-related properties used (in addition to van der Waals interactions) by ZRank.

### Comparison to the fuzzy oil drop model

**Figure 8:**
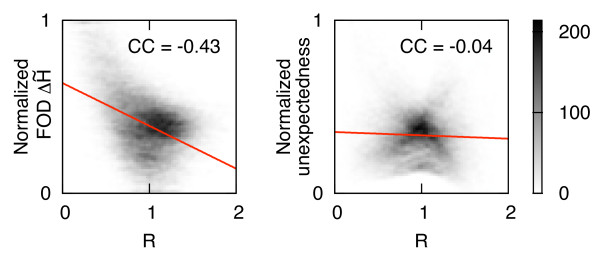
**Dependence of **$\Delta \tilde{H}$** and unexpectedness on reduced central distances**. Two normalized global estimators of hydrophobic excess or deficiency, $\Delta \tilde{H}$ (according to the fuzzy oil drop model) and the unexpectedness, vs. reduced distance from the geometric center of a protein, *R*. Linear fits and correlation coefficients (CC) for 2D histograms were calculated for all atoms of unbound proteins from the LB_48 _test set.

Ranking clusters according to the most unexpected atoms turned out to be less specific than the ordering based on the FOD-based discrepancy between theoretical and empirical hydrophobicity, $\Delta \tilde{H}$. Searching for the reason of disadvantageous cluster rankings we found that the FOD method not only quantifies the hydrophobicity discrepancy, but primarily indicates residues in the proximity to the molecular centroid (Figure 8). Visibly, the fuzzy oil drop model inadequately overestimates the hydrophobicity in protein cores. The satisfactory predictive capability and advantageous ranking of the FOD-based method can be explained by the observation that the distance to the centroid can be used autonomously for the detection of active sites and enzyme-ligand interfaces [73]. In our probabilistic approach, unexpectedness of atoms is virtually independent of their central distances.

### Availability

We developed a web server SurpResi for the prediction of functionally important sites based on the unusual central distances of atoms. The input of SurpResi server is a Protein Data Bank (PDB) file or user file in the PDB format. The output is a downloadable PDB file where the column of beta factors is replaced by the unexpectedness and the occupancy is replaced by the same value normalized to the range [0,1] over all protein atoms. In the header section, the file contains detailed information about clustering and ranking of clusters. The web server and source code are freely available at http://www.bioinformatics.org/surpresi.

## Discussion

The presented approach quantifies polar and directional propensities of amino acids using the partition in the knowledge-based continuous gradient of hydrophobicity generated by the protein itself. It yields a middle level of description of hydrophobic preferences between (coarse-grained) scales of hydrophobicity and (fine-grained) residue-residue contact matrices, where more specific local effects such as homophilic, counterion or phenyl rings interactions can be expressed explicitly [74]. It has been already demonstrated that reduced representations and global geometric potentials are capable of a quantitative description of protein-ligand binding sites [75,76].

The adopted view concentrates on the characterization of proteins not assuming any specific chemical properties of ligands. Although based on a statistical model parametrized assuming spherical shapes of proteins (resembling the assumption behind the generalized Born solvation model), the method works well for moderately aspherical macromolecules, allowing for not only descriptive but also predictive applications. We do not incorporate into the identification method any additional features, such as the solvent accessible area or evolutionary conservation; the direct distance to the centroid was used only for the ranking in order to enable fair comparison with the FOD method; our measure is assigned homogeneously and isotropically in the whole protein volume, thus allowing for the examination of the predictive potential of the sole unexpectedness.

Favorable outcomes of our approach, especially when applied to enzymatic active sites, can be explained by analyzing the consequences of the requirement of the precise and resolute positioning of a ligand (as the prerequisite for chemical specificity), which can be best fulfilled by the creation of a binding pocket [77]. The burial of (still accessible) charged amino acids or the exposure of (partially unburied) conjugated aromatic ones, which are essential from the point of view of the mechanisms of the catalytic reactions, are not commensurate with their general expected radial positions in the bulk protein body. Frequently, despite their indented locations, pocket residues cannot be predominantly apolar as well, because of the need for the presence of bound water molecules assisting the catalysis (involved in, e.g., nucleophilic attack).

The most unexpected atoms are usually found in the deep-set parts of the pockets. The atomic depth has been found to be correlated with residue conservation [78,79] (more conserved amino acids create more contacts), which provides the explanation for the overlap between the sets of unexpected and conserved residues. It has been found, based on electrostatics, that functional sites comprise the most destabilizing residues [18]. Similarly, the unexpected amino acids are those introducing a local hydrophobic mismatch, plausibly counterbalanced by the formation of salt bridges and hydrogen bonding. The relation of the unexpectedness to the electrostatics is not, however, as simple as in the case of the conservation: buried charged residues can be encountered occasionally. It has been also demonstrated that electrostatic and hydrophobic interactions may compete [80]. This interplay is important with respect to the desolvation energy. The ease of desolvation is strongly predictive of protein-binding interfaces [29] and influences intricately ligand binding affinities [81]. As the hydrophobic interactions are dominant at protein interfaces [82], indicated scattered residues at the surface likely coincide with the view of the small fraction of hot-spots, which account for the majority of the binding energy [83].

Our approach yielded sets of parameters for every atom in an amino acid of a given type that is similar to the construction of a hydrophobicity scale, because the amount of information needed to characterize a protein is linearly proportional to the length of its sequence. The introduction of information-theoretic interpretation of hydrophobicity distributions may lead to valuable insights [84]. One result of the meeting of hydrophobicity and information theory, especially noteworthy in this context, supports our approach by demonstrating improvements in contact potentials tailored to the compositional properties of the sequences of interest [85].

The "mixture model" used in Equation 3 may be tuned via the expectation-maximization procedure to better fit the idealized distribution of the mass in individual proteins. However, we observed no improvement in the performance of the predictions for tuned forms, probably due to the already balanced composition of hydrophobic and polar amino acids in proteins selected by nature [86]. In this view, it would be interesting to check whether sequences of disordered or unfoldable structures give "mixture models" that deviate significantly from compact atomic distributions. It seems to be possible to apply the method from the smoothed surface towards the protein interior to some depth, and in this way cover proteins of more irregular shapes, consequently surpassing the most severe limitation of the approach. The attempt would require, however, the inquiry into the structure of hydrophobic cores in elongated or bent proteins.

The method is expected to be applicable for the functional annotation of low resolution structures, e.g., those resulting from mature homology modeling pipelines. Crude estimates of unexpectedness may be advantageous over computational geometry-based methods requiring precise atomic coordinates of active sites, where residues or even whole loops undergo significant displacements, not obeying the classic lock-and-key model [87].

## Conclusion

We present an approach that captures orientational propensities of amino acids in globular proteins and offers a balanced description of their hydrophobic preferences. The description is created at the granularity of individual (amino acid-dependent types of) atoms but does not enumerate explicitly all possible interactions between them.

The approach is useful for the construction of a generic method that quantifies the unexpectedness of occurrences of individual atoms in a given distance from the geometric center of a protein. It turns out that the characteristics can be applied to the recognition of binding sites of both small ligands (enzymatic active sites) and other proteins (protein-protein interfaces).

## Authors' contributions

MK conceived of the study, implemented the method, carried out computations, analyzed results and wrote the manuscript.

## Supplementary Material

Additional file 1 **Protein chains in the learning set**. Click here for file

Additional file 2 **Geometric characteristics of the learning set and their dependencies**. Click here for file

Additional file 3 **The plot of the probability density function used in this work**. Click here for file

Additional file 4 **Parameters of atomic distributions**. Click here for file

Additional file 5 **Probability densities of C*^α ^*and distal side chain atoms of 20 amino acids**. Click here for file

Additional file 6 **Probability densities of C*^α ^*and distal side chain atoms of Cys**. Click here for file

Additional file 7 **Details on the efficiency of the SurpResi applied to the LB_48 _test set**. Click here for file

## References

[B1] Li YY Hou TJ Goddard WA Computational modeling of structure-function of G protein-coupled receptors with applications for drug design Curr Med Chem 2010 17 12 1167 80 10.2174/092986710790827807 20158474

[B2] Fiorucci S Zacharias M Binding site prediction and improved scoring during flexible protein-protein docking with ATTRACT Proteins 2010 78 15 3131 9 10.1002/prot.22808 20715290

[B3] Seffernick JL de Souza ML Sadowsky MJ Wackett LP Melamine deaminase and atrazine chlorohydrolase: 98 percent identical but functionally different J Bacteriol 2001 183 8 2405 10 10.1128/JB.183.8.2405-2410.2001 11274097 PMC95154

[B4] Ivanisenko VA Pintus SS Grigorovich DA Kolchanov NA PDBSiteScan: a program for searching for active, binding and posttranslational modification sites in the 3D structures of proteins Nucleic Acids Res 2004 W549 54 10.1093/nar/gkh439 PMC441577 15215447

[B5] Jambon M Imberty A Deléage G Geourjon C A new bioinformatic approach to detect common 3D sites in protein structures Proteins 2003 52 2 137 45 10.1002/prot.10339 12833538

[B6] Doppelt-Azeroual O Delfaud F Moriaud F de Brevern AG Fast and automated functional classification with MED-SuMo: an application on purinebinding proteins Protein Sci 2010 19 4 847 67 10.1002/pro.364 20162627 PMC2867024

[B7] Brylinski M Skolnick J A threading-based method (FINDSITE) for ligand-binding site prediction and functional annotation Proc Natl Acad Sci USA 2008 105 129 34 10.1073/pnas.0707684105 18165317 PMC2224172

[B8] Thangudu RR Tyagi M Shoemaker BA Bryant SH Panchenko AR Madej T Knowledge-based annotation of small molecule binding sites in proteins BMC Bioinformatics 2010 11 365 10.1186/1471-2105-11-365 20594344 PMC2909224

[B9] Laskowski RA SURFNET: a program for visualizing molecular surfaces, cavities, and intermolecular interactions J Mol Graph 1995 13 5 323 30, 307-8 10.1016/0263-7855(95)00073-9 8603061

[B10] Brady GP Jr Stouten PF Fast prediction and visualization of protein binding pockets with PASS J Comput Aided Mol Des 2000 14 4 383 401 10.1023/A:1008124202956 10815774

[B11] Levitt DG Banaszak LJ POCKET: a computer graphics method for identifying and displaying protein cavities and their surrounding amino acids J Mol Graph 1992 10 4 229 34 10.1016/0263-7855(92)80074-N 1476996

[B12] Hendlich M Rippmann F Barnickel G LIGSITE: automatic and efficient detection of potential small molecule-binding sites in proteins J Mol Graph Model 1997 15 6 359 63, 389 10.1016/S1093-3263(98)00002-3 9704298

[B13] Weisel M Proschak E Schneider G PocketPicker: analysis of ligand binding-sites with shape descriptors Chem Cent J 2007 1 7 10.1186/1752-153X-1-7 17880740 PMC1994066

[B14] Liang J Edelsbrunner H Woodward C Anatomy of protein pockets and cavities: measurement of binding site geometry and implications for ligand design Protein Sci 1998 7 9 1884 97 10.1002/pro.5560070905 9761470 PMC2144175

[B15] Le Guilloux V Schmidtke P Tuffery P Fpocket: an open source platform for ligand pocket detection BMC Bioinformatics 2009 10 168 10.1186/1471-2105-10-168 19486540 PMC2700099

[B16] Coleman RG Sharp KA Protein pockets: inventory, shape, and comparison J Chem Inf Model 2010 50 4 589 603 10.1021/ci900397t 20205445 PMC2859996

[B17] Yuan Z Zhao J Wang ZX Flexibility analysis of enzyme active sites by crystallographic temperature factors Protein Eng 2003 16 2 109 14 10.1093/proeng/gzg014 12676979

[B18] Elcock AH Prediction of functionally important residues based solely on the computed energetics of protein structure J Mol Biol 2001 312 4 885 96 10.1006/jmbi.2001.5009 11575940

[B19] Bate P Warwicker J Enzyme/non-enzyme discrimination and prediction of enzyme active site location using charge-based methods J Mol Biol 2004 340 2 263 76 10.1016/j.jmb.2004.04.070 15201051

[B20] Laurie ATR Jackson RM Q-SiteFinder: an energy-based method for the prediction of protein-ligand binding sites Bioinformatics 2005 21 9 1908 16 10.1093/bioinformatics/bti315 15701681

[B21] Brylinski M Prymula K Jurkowski W Kochańczyk M Stawowczyk E Konieczny L Roterman I Prediction of functional sites based on the fuzzy oil drop model PLoS Comput Biol 2007 3 5 e94 10.1371/journal.pcbi.0030094 17530916 PMC1876487

[B22] Oda A Yamaotsu N Hirono S Evaluation of the searching abilities of HBOP and HBSITE for binding pocket detection J Comput Chem 2009 30 16 2728 37 10.1002/jcc.21299 19399761

[B23] Bagley SC Altman RB Characterizing the microenvironment surrounding protein sites Protein Sci 1995 4 4 622 35 7613462 10.1002/pro.5560040404 PMC2143108

[B24] Jones S Thornton JM Prediction of protein-protein interaction sites using patch analysis J Mol Biol 1997 272 133 43 10.1006/jmbi.1997.1233 9299343

[B25] Ondrechen MJ Clifton JG Ringe D THEMATICS: a simple computational predictor of enzyme function from structure Proc Natl Acad Sci USA 2001 98 22 12473 8 10.1073/pnas.211436698 11606719 PMC60078

[B26] Bordner AJ Predicting small ligand binding sites in proteins using backbone structure Bioinformatics 2008 24 24 2865 71 10.1093/bioinformatics/btn543 18940825 PMC2639300

[B27] Cilia E Passerini A Automatic prediction of catalytic residues by modeling residue structural neighborhood BMC Bioinformatics 2010 11 115 10.1186/1471-2105-11-115 20199672 PMC2844391

[B28] Panjkovich A Daura X Assessing the structural conservation of protein pockets to study functional and allosteric sites: implications for drug discovery BMC Struct Biol 2010 10 9 10.1186/1472-6807-10-9 20356358 PMC2864279

[B29] Burgoyne NJ Jackson RM Predicting protein interaction sites: binding hot-spots in protein-protein and protein-ligand interfaces Bioinformatics 2006 22 11 1335 42 10.1093/bioinformatics/btl079 16522669

[B30] Tong W Wei Y Murga LF Ondrechen MJ Williams RJ Partial order optimum likelihood (POOL): maximum likelihood prediction of protein active site residues using 3D structure and sequence properties PLoS Comput Biol 2009 5 e1000266 10.1371/journal.pcbi.1000266 19148270 PMC2612599

[B31] Capra JA Laskowski RA Thornton JM Singh M Funkhouser TA Predicting protein ligand binding sites by combining evolutionary sequence conservation and 3D structure PLoS Comput Biol 2009 5 12 e1000585 10.1371/journal.pcbi.1000585 19997483 PMC2777313

[B32] Huang B Schroeder M LIGSITEcsc: predicting ligand binding sites using the Connolly surface and degree of conservation BMC Struct Biol 2006 6 19 10.1186/1472-6807-6-19 16995956 PMC1601958

[B33] Bray T Chan P Bougouffa S Greaves R Doig AJ Warwicker J SitesIdentify: a protein functional site prediction tool BMC Bioinformatics 2009 10 379 10.1186/1471-2105-10-379 19922660 PMC2783165

[B34] Laskowski RA Watson JD Thornton JM ProFunc: a server for predicting protein function from 3D structure Nucleic Acids Res 2005 W89 93 10.1093/nar/gki414 PMC1160175 15980588

[B35] Huang B MetaPocket: a meta approach to improve protein ligand binding site prediction OMICS 2009 13 4 325 30 10.1089/omi.2009.0045 19645590

[B36] Brylinski M Kochańczyk M Konieczny L Roterman I Sequence-structure-function relation characterized in silico In Silico Biol 2006 6 6 589 600 17518766

[B37] Jones S Thornton JM Analysis of protein-protein interaction sites using surface patches J Mol Biol 1997 272 121 32 10.1006/jmbi.1997.1234 9299342

[B38] Konieczny L Brylinski M Roterman I Gauss-function-based model of hydrophobicity density in proteins In Silico Biol 2006 6 1-2 15 22 16789910

[B39] Gomes ALC de Rezende JR Pereira de Araújo AF Shakhnovich EI Description of atomic burials in compact globular proteins by Fermi-Dirac probability distributions Proteins 2007 66 2 304 20 17109406 10.1002/prot.21137

[B40] Kauzmann W Some factors in the interpretation of protein denaturation Adv Protein Chem 1959 14 1 63 14404936 10.1016/s0065-3233(08)60608-7

[B41] Richards FM Lim WA An analysis of packing in the protein folding problem Q Rev Biophys 1993 26 4 423 98 10.1017/S0033583500002845 8058892

[B42] Dill KA Dominant forces in protein folding Biochemistry 1990 29 31 7133 55 10.1021/bi00483a001 2207096

[B43] Rackovsky S Scheraga HA Hydrophobicity, hydrophilicity, and the radial and orientational distributions of residues in native proteins Proc Natl Acad Sci USA 1977 74 12 5248 51 10.1073/pnas.74.12.5248 271950 PMC431666

[B44] Jha AN Vishveshwara S Banavar JR Amino acid interaction preferences in proteins Protein Sci 2010 19 3 603 16 10.1002/pro.339 20073083 PMC2866284

[B45] Nishikawa K Ooi T Correlation of the amino acid composition of a protein to its structural and biological characters J Biochem 1982 91 5 1821 4 7096320 10.1093/oxfordjournals.jbchem.a133877

[B46] Taguchi Yh Gromiha MM Application of amino acid occurrence for discriminating different folding types of globular proteins BMC Bioinformatics 2007 8 404 10.1186/1471-2105-8-404 17953741 PMC2174517

[B47] Ma BG Chen LL Zhang HY What determines protein folding type? An investigation of intrinsic structural properties and its implications for understanding folding mechanisms J Mol Biol 2007 370 3 439 48 10.1016/j.jmb.2007.04.051 17524416

[B48] Rackovsky S Global characteristics of protein sequences and their implications Proc Natl Acad Sci USA 2010 107 19 8623 6 10.1073/pnas.1001299107 20421501 PMC2889366

[B49] Roy S Martinez D Platero H Lane T Werner-Washburne M Exploiting amino acid composition for predicting protein-protein interactions PLoS One 2009 4 11 e7813 10.1371/journal.pone.0007813 19936254 PMC2775920

[B50] Berman HM Westbrook J Feng Z Gilliland G Bhat TN Weissig H Shindyalov IN Bourne PE The Protein Data Bank Nucleic Acids Res 2000 28 235 42 10.1093/nar/28.1.235 10592235 PMC102472

[B51] Baumgärtner A Shapes of flexible vesicles at constant volume J Chem Phys 1993 98 7496 7501 10.1063/1.464689

[B52] Galzitskaya OV Bogatyreva NS Ivankov DN Compactness determines protein folding type J Bioinform Comput Biol 2008 6 4 667 80 10.1142/S0219720008003618 18763735

[B53] Murzin AG Brenner SE Hubbard T Chothia C SCOP: a structural classification of proteins database for the investigation of sequences and structures J Mol Biol 1995 247 4 536 40 7723011 10.1006/jmbi.1995.0159

[B54] Orengo CA Michie AD Jones S Jones DT Swindells MB Thornton JM CATH - a hierarchic classification of protein domain structures Structure 1997 5 8 1093 108 10.1016/S0969-2126(97)00260-8 9309224

[B55] Li W Jaroszewski L Godzik A Clustering of highly homologous sequences to reduce the size of large protein databases Bioinformatics 2001 17 3 282 3 10.1093/bioinformatics/17.3.282 11294794

[B56] Brylinski M Kochanczyk M Broniatowska E Roterman I Localization of ligand binding site in proteins identified in silico J Mol Model 2007 13 6-7 665 75 10.1007/s00894-007-0191-x 17394030

[B57] Arteca GA Scaling behavior of some molecular shape descriptors of polymer chains and protein backbones Phys Rev E 1994 49 3 2417 2428 10.1103/PhysRevE.49.2417 9961485

[B58] Porter CT Bartlett GJ Thornton JM The Catalytic Site Atlas: a resource of catalytic sites and residues identified in enzymes using structural data Nucleic Acids Res 2004 D129 33 10.1093/nar/gkh028 PMC308762 14681376

[B59] Sanner MF Olson AJ Spehner JC Reduced surface: an efficient way to compute molecular surfaces Biopolymers 1996 38 3 305 20 10.1002/(SICI)1097-0282(199603)38:3<305::AID-BIP4>3.0.CO;2-Y 8906967

[B60] Hwang H Vreven T Janin J Weng Z Protein-protein docking benchmark version 4.0 Proteins 2010 78 15 3111 4 10.1002/prot.22830 20806234 PMC2958056

[B61] Mintseris J Pierce B Wiehe K Anderson R Chen R Weng Z Integrating statistical pair potentials into protein complex prediction Proteins 2007 69 3 511 20 10.1002/prot.21502 17623839

[B62] Pierce B Weng Z ZRANK: reranking protein docking predictions with an optimized energy function Proteins 2007 67 4 1078 86 10.1002/prot.21373 17373710

[B63] Li L Guo D Huang Y Liu S Xiao Y ASPDock: protein-protein docking algorithm using atomic solvation parameters model BMC Bioinformatics 2011 12 36 10.1186/1471-2105-12-36 21269517 PMC3039575

[B64] Gabb HA Jackson RM Sternberg MJ Modelling protein docking using shape complementarity, electrostatics and biochemical information J Mol Biol 1997 272 106 20 10.1006/jmbi.1997.1203 9299341

[B65] Hwang H Pierce B Mintseris J Janin J Weng Z Protein-protein docking benchmark version 3.0 Proteins 2008 73 3 705 9 10.1002/prot.22106 18491384 PMC2726780

[B66] Glaser F Pupko T Paz I Bell RE Bechor-Shental D Martz E Ben-Tal N ConSurf: identification of functional regions in proteins by surface-mapping of phylogenetic information Bioinformatics 2003 19:163 4 10.1093/bioinformatics/19.1.163 12499312

[B67] Eisenberg D Weiss RM Terwilliger TC The hydrophobic moment detects periodicity in protein hydrophobicity Proc Natl Acad Sci USA 1984 81 140 4 10.1073/pnas.81.1.140 6582470 PMC344626

[B68] Wu S Liu T Altman RB Identification of recurring protein structure microenvironments and discovery of novel functional sites around CYS residues BMC Struct Biol 2010 10 4 10.1186/1472-6807-10-4 20122268 PMC2833161

[B69] Marino SM Gladyshev VN Cysteine function governs its conservation and degeneration and restricts its utilization on protein surfaces J Mol Biol 2010 404 5 902 16 10.1016/j.jmb.2010.09.027 20950627 PMC3061813

[B70] Klotz IM Comparison of molecular structures of proteins: helix content; distribution of apolar residues Arch Biochem Biophys 1970 138 2 704 6 10.1016/0003-9861(70)90401-7 4988452

[B71] Lins L Thomas A Brasseur R Analysis of accessible surface of residues in proteins Protein Sci 2003 12 7 1406 17 10.1110/ps.0304803 12824487 PMC2323943

[B72] Meirovitch H Rackovsky S Scheraga HA Empirical studies of hydrophobicity. 1. Effect of protein size on the hydrophobic behavior of amino acids Macromolecules 1980 13 6 1398 1405 10.1021/ma60078a013

[B73] Ben-Shimon A Eisenstein M Looking at enzymes from the inside out: the proximity of catalytic residues to the molecular centroid can be used for detection of active sites and enzyme-ligand interfaces J Mol Biol 2005 351 2 309 26 10.1016/j.jmb.2005.06.047 16019028

[B74] Singer MS Vriend G Bywater RP Prediction of protein residue contacts with a PDB-derived likelihood matrix Protein Eng 2002 15 9 721 5 10.1093/protein/15.9.721 12456870

[B75] Xie L Bourne PE A robust and efficient algorithm for the shape description of protein structures and its application in predicting ligand binding sites BMC Bioinformatics 2007 8 Suppl 4 S9 10.1186/1471-2105-8-S4-S9 17570152 PMC1892088

[B76] Feldman HJ Labute P Pocket similarity: are alpha carbons enough? J Chem Inf Model 2010 50 8 1466 75 10.1021/ci100210c 20690656

[B77] Campbell SJ Gold ND Jackson RM Westhead DR Ligand binding: functional site location, similarity and docking Curr Opin Struct Biol 2003 13 3 389 95 10.1016/S0959-440X(03)00075-7 12831892

[B78] Godzik A Sander C Conservation of residue interactions in a family of Ca-binding proteins Protein Eng 1989 2 8 589 96 10.1093/protein/2.8.589 2813336

[B79] Pintar A Carugo O Pongor S Atom depth in protein structure and function Trends Biochem Sci 2003 28 11 593 7 10.1016/j.tibs.2003.09.004 14607089

[B80] Wang L Friesner RA Berne BJ Competition of electrostatic and hydrophobic interactions between small hydrophobes and model enclosures J Phys Chem B 2010 114 21 7294 301 10.1021/jp100772w 20443643 PMC3040037

[B81] Wang L Berne BJ Friesner RA Ligand binding to protein-binding pockets with wet and dry regions Proc Natl Acad Sci USA 2011 108 4 1326 30 10.1073/pnas.1016793108 21205906 PMC3029693

[B82] Jones S Thornton JM Principles of protein-protein interactions Proc Natl Acad Sci USA 1996 93 13 20 10.1073/pnas.93.1.13 8552589 PMC40170

[B83] Tuncbag N Gursoy A Keskin O Identification of computational hot spots in protein interfaces: combining solvent accessibility and inter-residue potentials improves the accuracy Bioinformatics 2009 25 12 1513 20 10.1093/bioinformatics/btp240 19357097

[B84] Pereira de Araujo AF Onuchic JN A sequence-compatible amount of native burial information is sufficient for determining the structure of small globular proteins Proc Natl Acad Sci USA 2009 106 45 19001 4 10.1073/pnas.0910851106 19858496 PMC2776435

[B85] Solis AD Rackovsky SR Information-theoretic analysis of the reference state in contact potentials used for protein structure prediction Proteins 2010 78 6 1382 97 20034109 10.1002/prot.22652 PMC2841228

[B86] Bastolla U Porto M Roman HE Vendruscolo M Principal eigenvector of contact matrices and hydrophobicity profiles in proteins Proteins 2005 58 22 30 15523667 10.1002/prot.20240

[B87] Schmidt A Lamzin VS Internal motion in protein crystal structures Protein Sci 2010 19 5 944 53 20198682 10.1002/pro.371 PMC2868237

[B88] Rose GD Geselowitz AR Lesser GJ Lee RH Zehfus MH Hydrophobicity of amino acid residues in globular proteins Science 1985 229 4716 834 8 10.1126/science.4023714 4023714

[B89] Naderi-Manesh H Sadeghi M Arab S Moosavi Movahedi AA Prediction of protein surface accessibility with information theory Proteins 2001 42 4 452 9 10.1002/1097-0134(20010301)42:4<452::AID-PROT40>3.0.CO;2-Q 11170200

[B90] Biou V Gibrat JF Levin JM Robson B Garnier J Secondary structure prediction: combination of three different methods Protein Eng 1988 2 3 185 91 10.1093/protein/2.3.185 3237683

[B91] Cornette JL Cease KB Margalit H Spouge JL Berzofsky JA DeLisi C Hydrophobicity scales and computational techniques for detecting amphipathic structures in proteins J Mol Biol 1987 195 3 659 85 10.1016/0022-2836(87)90189-6 3656427

[B92] Guy HR Amino acid side-chain partition energies and distribution of residues in soluble proteins Biophys J 1985 47 61 70 10.1016/S0006-3495(85)83877-7 3978191 PMC1435068

[B93] Wilce MCJ Aguilar MI Hearn MTW Physicochemical basis of amino acid hydrophobicity scales: evaluation of four new scales of amino acid hydrophobicity coefficients derived from RP-HPLC of peptides Analytical Chemistry 1995 67 7 1210 1219 10.1021/ac00103a012

