# Supplementary material for: Prediction of functionally important residues in globular proteins from unusual central distances of amino acids
Source: BMC Struct Biol. 2011 Sep 18;11:34. doi: 10.1186/1472-6807-11-34 (PMC3188475; doi:10.1186/1472-6807-11-34)
Supplement: Additional file 1 — Protein chains in the learning set. [file 1472-6807-11-34-S1.PDF]

Table S1. PDB ids of 775 globular chains in the non-redundant learning set derived in this work.

1c3d_A	1wka_A	1kcq_A	2p3k_A	1d7p_M	1gv8_A	2vw5_A	1czs_A	1b1c_A	1eur_A	1ea5_A
1knb_A	1t1i_A	1thg_A	1srv_A	1gz7_A	1h49_A	2ri9_A	1m21_B	1myr_A	1wdp_A	2f6d_A
1nxc_A	1s95_A	2j75_B	1wcg_B	1hxn_A	1cge_A	1kex_A	1h10_A	1uuq_A	1ug6_A	2v3r_A
1ksc_A	1h46_X	1jdw_A	6cp4_A	1bn8_A	1h4p_B	1xwt_A	1ojj_B	1eqc_A	2fpv_A	1xnc_A
1t64_B	1r3r_A	1enu_A	1uqy_A	1dim_A	1edg_A	1r87_A	2d5j_A	1c3p_A	2aba_A	2d8l_A
966c_A	1f2j_A	3eau_A	1qjw_B	1eyw_A	2hu6_A	1xx2_A	1ocj_A	1g01_A	1vfl_A	1cem_A
1jta_A	1rgy_A	1ke4_B	1idk_A	1w3h_B	1qcx_A	1h6l_A	1xkn_A	1qaz_A	1a4m_A	1pw8_A
1w0h_A	1chd_A	1ezw_A	1fhw_A	1xyz_B	1lqa_B	2o0m_A	1qnp_A	1y65_A	1onr_B	1r66_A
1xfk_A	1j8t_A	1fob_A	1gxm_B	1hjs_A	1qjf_A	1n82_B	1ry8_A	1rhc_A	1g7f_A	2d2j_A
1jk7_A	1o3y_A	1vbr_B	2ghs_A	1mrq_A	1ds0_A	2gvv_A	1zpg_C	1v71_A	1lzl_A	1q5m_B
1rqj_A	2pll_B	1ppo_A	4lip_D	2iki_A	1gyh_A	1zgk_A	1tca_A	1qwk_A	1gmy_A	1frb_A
1l9x_C	1fhd_A	1up0_A	2hxz_B	1wl7_A	3c9e_A	1mlw_A	1v0k_A	1ute_A	1pyf_A	2cyg_A
1gx4_A	2had_A	2ixt_A	1rkd_A	1hdu_A	5a3h_A	1lyv_A	2p4o_A	1h1n_A	2p41_A	1cqw_A
1gok_A	1dqy_A	1g4h_A	1bqc_A	1nq6_A	1lok_A	1cnv_A	1q0z_A	1wb4_B	1ltu_A	1jln_A
1hq0_A	1ls6_A	2gmn_A	1nnh_A	1mtz_A	1uv4_A	1u5h_A	1bf6_A	1n57_A	1eok_A	1nar_A
1b8o_A	2i47_A	1o4y_A	1pzt_A	1tml_A	1fqg_A	1q7f_A	1f0n_A	1qtw_A	1tkj_A	1ukb_A
1y21_A	1a2q_A	1r88_B	2pkc_A	1va4_A	1hl7_B	1thm_A	1umz_B	1i6n_A	2gu5_B	1qqf_A
1brt_A	1deu_A	1wma_A	1om0_A	1ja9_A	1a8q_A	1llo_A	1a8s_A	1dyp_A	1ak0_A	1jov_A
3b68_A	2nv6_A	1sml_A	1st3_A	1tib_A	3tgl_A	1jjf_A	1ako_A	1pa7_A	1j6o_A	1j02_A
1fsf_A	2gnp_A	1tys_A	2a0n_A	1lug_A	1qhv_A	2cdd_A	1a28_A	1thf_D	1ny1_B	2pbl_B
1jfr_B	1vic_A	1b0u_A	1o0x_A	2f1n_A	1sep_A	1uza_A	1xfj_A	1v9e_A	1p1x_A	2nmx_B
1k77_A	1txo_A	1rv9_A	1zzm_A	1oha_A	1m33_A	3c70_A	2bwa_A	1xa1_B	1ax2_A	1h70_A
1caq_A	1jyk_A	1b2l_A	1vc4_B	2q46_A	1x06_A	1gqn_A	1km3_A	2gpu_A	1zk0_A	1n55_A
1g24_A	1qwg_A	2o34_A	1e58_A	1lyx_A	1qrl_A	1jzt_A	1pbt_A	1udh_A	1rw7_A	1mve_A
1i9t_A	1qo2_A	1vyb_B	2qfo_B	1o1y_A	1ini_A	1nxd_3	1din_A	1mvq_A	1fx2_A	1jg4_A
2nlr_A	1xjz_A	1k4l_A	1dex_A	1wab_A	1fj2_B	1fx4_A	1eug_A	1jjt_A	1dxk_A	1g61_B
1g6c_A	1nfp_A	1kdt_A	2abw_B	2pof_A	1gxy_A	1i1n_A	1upi_A	1dak_A	1uu6_A	1uai_A
1u9c_A	2cl5_B	2hxm_A	3b5e_B	1njs_B	1oq1_C	1okb_A	1oa4_A	1k7j_A	1aec_A	1q7r_A
1uol_B	1q0u_A	1oa2_F	2o2x_A	1fva_A	1l8b_B	1nn1_A	3c7i_A	1jfx_A	1txl_A	1wnx_B
1g3u_A	2ayh_A	1agy_A	1r55_A	1l8f_A	1gbg_A	1ijb_A	1cpn_A	1pt6_B	4tmk_A	4eng_A
3gar_A	1v77_A	1ppn_A	1hbp_A	1ff3_C	1yzq_A	1hjz_B	1h4h_D	1p3u_A	1aun_A	1dix_A
1o0e_A	1pzs_A	1d4o_A	1h2e_A	1vg8_A	2blu_A	1nf8_A	1cjw_A	1qoz_B	1bs9_A	2cd2_A
1he4_A	1u8y_B	1ui0_A	1lhu_A	1jm1_A	1vk2_A	1kuf_A	1ukz_A	1nwa_A	1nd1_A	1h4e_A
1vp8_A	2cdn_A	1iqq_A	1wc9_A	1f5j_A	2gf0_A	2i6g_B	1e87_A	1tf1_B	1z06_A	1p5f_A
1bsw_A	1m55_B	1j1f_A	1ido_A	1xnk_B	1rie_A	1atz_B	1kmq_A	2atv_A	1ioo_A	1g5t_A
1el4_A	1x3s_A	2nr7_A	1jf0_A	1qf9_A	1tc5_D	1lm4_A	1yna_A	1dus_A	1j54_A	1pvx_A
2pth_A	1ia1_B	1mh1_A	1oqv_A	1uxo_A	2isb_A	1uhh_B	2o7n_A	1sl8_A	1oix_A	1pl3_B
2dfb_A	1hzt_A	8dfr_A	1ihc_A	1qra_A	1i8a_A	1eq6_A	1vkf_C	1bsz_B	1koe_A	1mvt_A
1eyl_A	1beh_A	1jfu_A	1l1q_A	2if6_A	1gbs_A	1pmh_X	1u17_B	1epz_A	1r8n_A	1ywd_A
2dfn_A	2nn5_A	1lqy_A	1nxj_B	1x1r_A	1kn3_A	1ky2_A	1h0p_A	2i6c_A	2fn4_A	2qxu_H
1mr3_F	1fzq_A	1n5n_B	1tiq_A	2eu7_X	1j83_B	1vjf_A	1rxd_B	1eiz_A	1im5_A	1i06_A
2ot9_A	1jwq_A	1oh4_A	1yzl_A	1iko_P	1znk_A	1a58_A	2hia_A	1ghe_B	2a2n_C	1gwy_B
1i6t_A	1qfv_B	1vhs_A	1wba_A	1vhh_A	1sl5_A	1vi4_A	1cv8_A	1euj_A	1qmy_A	2cyh_A
2fko_A	1dyw_A	1h4o_C	1zmf_A	2fcr_A	1mmq_A	2ery_B	2c8s_A	2ow9_A	1jhj_A	1n6n_A
2bem_C	1ek0_A	1vi3_A	1yvd_A	1ofv_A	1obo_B	1rm8_A	1mug_A	2cua_A	1f3z_A	1z4r_A
1z2a_A	1od3_A	1ddw_A	1uuy_A	1kao_A	2nvh_A	2gkp_A	1xo7_A	1qst_A	1vai_A	1nyk_A
2bit_X	1zp5_A	1g81_A	1nrz_D	1dly_A	1sen_A	1mz4_A	1n08_B	1uz2_X	1ist_B	1gpr_A
3dfr_A	1tp9_C	1htw_A	1d2a_A	2ijq_A	2icg_A	1mxi_A	1fm4_A	1mfm_A	1ra8_A	1dg7_A
1q0n_A	1xdf_B	1edu_A	2hbo_A	1dg9_A	1jyh_A	1e00_A	2fqt_A	1elk_A	1kva_A	1kng_A
1bj7_A	1gy1_A	1npk_A	1bfg_A	1ab0_A	2oeb_A	1o1x_A	1icx_A	1m16_B	1gui_A	2spo_A
1oj6_D	1yaz_A	1o7u_A	1md6_A	1emy_A	1mno_B	2nsr_A	1gdj_A	1gwm_A	2ob5_A	1l1d_B
1lic_A	1q0e_A	1fg4_A	1id0_A	1oal_A	1w1g_A	2i8g_A	1e5p_B	1st9_A	1stn_A	1oz9_A
1zzo_A	1nb9_A	1akt_A	1t2w_C	1mba_A	1h97_B	1jf4_A	1o4w_A	1kjl_A	1it2_A	2oyn_A
2hd9_A	1uy3_A	1p90_A	1at0_A	2d59_A	1lit_A	1q1u_A	1j7g_A	1b20_A	1tzx_B	1w9t_A
1hdk_A	1gz2_A	1iuk_A	1ov8_B	1rfs_A	1fvx_A	1ktg_A	1lhi_A	1jer_A	2fs6_B	3bzp_A
1c1f_A	1xs0_A	1eca_A	1o13_A	1mvo_A	1moy_A	1pdo_A	1lu4_A	2aif_A	1is6_A	3gal_A
1vyf_A	1p0z_A	1dqg_A	1e29_A	1r9h_A	1fsj_B	1opb_C	1uc7_B	1tu9_A	2fuf_A	1o8v_A
2ia7_A	1kqw_A	1zwz_A	1bea_A	1c52_A	1uxx_X	2ohw_B	1c7k_A	1srr_A	1icm_A	1hmt_A
1mdc_A	1lju_A	3nul_A	1jb3_A	1mai_A	1wna_A	1cc3_A	1mc9_A	1r29_A	2czw_A	1chn_A
1cuo_A	2ccw_A	1ow4_B	1u79_A	1cot_A	1a4a_B	1ou8_B	1i3u_A	1u29_A	2bt6_A	1ijt_A
1tp6_A	2fi9_A	1doi_A	1ijx_A	1zes_A	1rzy_A	1dbw_B	2gte_B	1fao_A	1t1j_A	1ugu_A
1jug_A	1r26_A	1eaz_A	8paz_A	1cxc_A	2cw4_A	1oae_A	1zia_A	1m5t_A	1rtx_A	1f9m_A
2fc3_A	1v30_A	1c44_A	2trx_A	1hq8_A	1hxr_B	1upq_A	1pmy_A	1wou_A	1ufy_A	1f7l_A
1whi_A	1qto_A	1m9z_A	3b7c_A	2pl1_A	1buo_A	1ifr_A	2a9o_A	1tmy_A	1ikt_A	1ra4_A
1tq3_A	1o7i_B	1opc_A	2cyj_A	1h4y_B	1h8u_A	2fne_B	2byg_A	2od5_A	4fiv_A	1gou_B
1pz4_A	1dlw_A	1mg4_A	6fiv_A	1td0_D	1thx_A	2pyq_A	1rtu_A	1svy_A	2iay_A	1o4i_A
1n8v_B	1dw0_B	1ytc_A	3c2c_A	2q3w_A	1ccr_A	2o3f_C	1i7h_C	1qwx_B	1pva_A	1kr7_A
1rwy_A	1n9l_A	5pal_A	1a75_B	2dg3_A	1tuw_A	1b8r_A	1bkr_A	1rwy_B	1bu3_A	1irv_A
1kaf_B	1omd_A	1gn0_A	1i1j_B	1d3w_A	1co6_A	2q5b_B	1iib_B	1oqq_B	2r48_A	1erw_A
1t5k_B	1rms_A	1hrc_A	2fmb_A	1i0x_D	1ln4_A	5cyt_R	2h3l_B	1xmt_A	1h7m_A	1tsf_A
4vub_A	2bo1_A	1l8r_A	1o5u_A	1n3y_A						
