# Supplementary material for: Prediction of functionally important residues in globular proteins from unusual central distances of amino acids
Source: BMC Struct Biol. 2011 Sep 18;11:34. doi: 10.1186/1472-6807-11-34 (PMC3188475; doi:10.1186/1472-6807-11-34)

Figure S1. Histograms and dependencies of several characteristics of the learning protein set (CC – correlation coefficient).

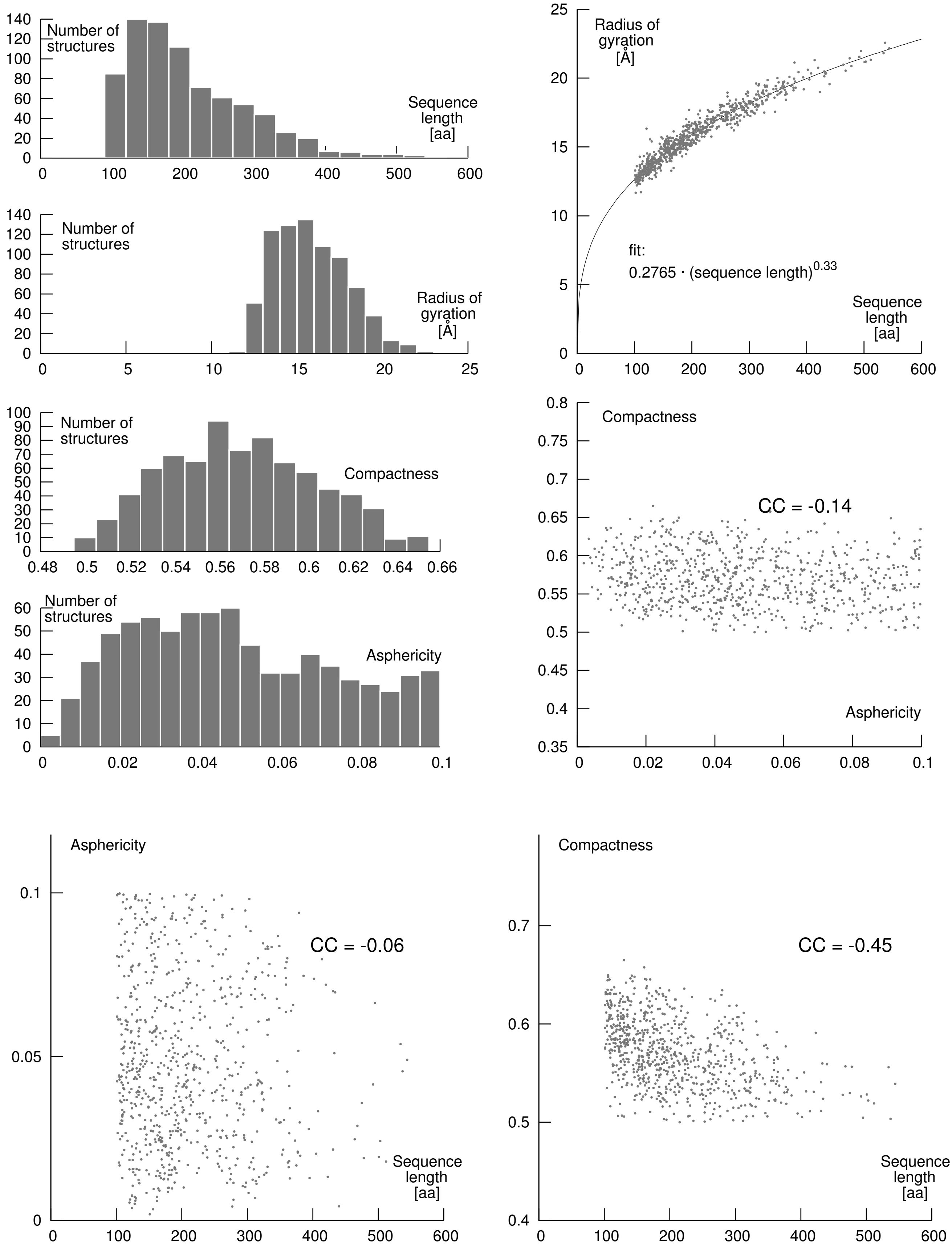

Supplement: Additional file 2 — Geometric characteristics of the learning set and their dependencies. [file 1472-6807-11-34-S2.PDF]
