# Supplementary material for: Prediction of functionally important residues in globular proteins from unusual central distances of amino acids
Source: BMC Struct Biol. 2011 Sep 18;11:34. doi: 10.1186/1472-6807-11-34 (PMC3188475; doi:10.1186/1472-6807-11-34)
Supplement: Additional file 3 — The plot of the probability density function used in this work. [file 1472-6807-11-34-S3.PDF]

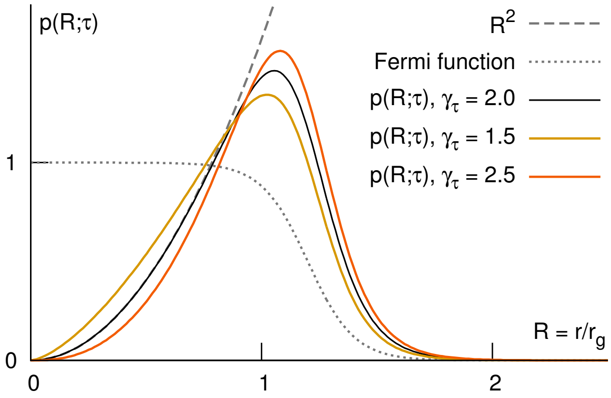

Figure S2. Probability density function used in this work:

$$p(R;\tau) = \frac{A_\tau\, R^{\gamma_\tau}}{1+\exp(\beta_\tau(R-\mu_\tau))}$$

for example values of γ_τ. Parameters: $\mu_\tau = 1.2$, $\beta_\tau = 10$; values of A_τ are chosen accordingly to normalize distributions. In general, when $\gamma_\tau < 2$, the function better fits histograms of atomic central distances for hydrophobic amino acids; when $\gamma_\tau > 2$, it better fits histograms of atomic distances for hydrophilic residues. For $\gamma_\tau = 2$ the function adopts the simplest form of a special case ($\alpha_\tau = 1$) demonstrated by Gomes et al. (*Proteins* 66(2):304-20 (2007)). The location of the maximum is $\beta^{-1}\,(\gamma + W(\gamma\exp(\beta\mu-\gamma)))$, where W is the (Lambert's) omega function, and the mean value can be estimated by $-A\beta^{-(\gamma+2)}\,\Gamma(\gamma+2)\,\mathrm{Li}_{\gamma+2}\,(-\exp(\beta\mu))$, where Γ and Li are the (Euler's) gamma and (Jonquière's) polylogarithm functions.
