# Supplementary material for: Prediction of functionally important residues in globular proteins from unusual central distances of amino acids
Source: BMC Struct Biol. 2011 Sep 18;11:34. doi: 10.1186/1472-6807-11-34 (PMC3188475; doi:10.1186/1472-6807-11-34)
Supplement: Additional file 4 — Parameters of atomic distributions. [file 1472-6807-11-34-S4.PDF]

Table S2. Parameters of the probability density function: $p(R;\tau) = \frac{A_\tau R^{\gamma_\tau}}{1+\exp(\beta_\tau(R-\mu_\tau))}$ derived from histograms of reduced central distances, R, collected for all types of heavy atoms of all types of amino acids as ocurring in globular proteins from the learning set (listed in the supplementary Table S1). Heavy atoms in side chains that are characterized by central distances distributed significantly different from central distances of C^α atoms (Kolmogorov-Smirnov tests with p-value < 0.000001) were marked with stars.

τ=(Aa,	Atom)	A_τ	μ_τ	β_τ	γ_τ
A	N	1.85954	1.14344	9.62103	2.01663
A	O	1.88563	1.14069	9.13446	2.1443
A	C	1.919	1.13577	9.5249	2.12155
A	C^α	1.86656	1.13408	8.70091	1.99614
· A	C^β	1.90715	1.10367	7.09949	1.9342
C_{SS}	N	2.42082	1.07441	8.54337	3.0098
C_{SS}	O	2.16193	1.11001	8.78373	2.88278
C_{SS}	C	2.22361	1.10463	9.01742	2.9227
C_{SS}	C^α	2.4535	1.06968	8.54859	2.96551
· C_{SS}	C^β	3.61427	0.969087	8.10373	3.65727
· C_{SS}	S^γ	4.87096	0.895388	7.75633	3.96847
C_{SH}	N	2.24242	1.01742	9.4792	1.49511
C_{SH}	O	2.03475	1.0501	9.11637	1.43533
C_{SH}	C	2.06666	1.04238	9.39009	1.41924
C_{SH}	C^α	2.51755	0.980198	8.93293	1.60296
· C_{SH}	C^β	2.69044	0.953012	8.91117	1.59813
· C_{SH}	S^γ	2.98544	0.92291	8.7473	1.68042
C	N	2.20509	1.0562	8.49479	1.85681
C	O	2.0752	1.09025	8.52757	1.80324
C	C	2.03371	1.08615	8.90352	1.78213
C	C^α	2.42883	1.02375	8.19802	1.95379
· C	C^β	2.77991	0.979176	7.9176	2.05535
· C	S^γ	3.06552	0.947826	7.78738	2.11068
D	N	1.85486	1.18094	11.289	3.27425
D	O	1.89458	1.1661	10.1346	3.40543
D	C	1.89924	1.17198	10.967	3.40343
D	C^α	1.74977	1.19626	11.1195	3.43601
* D	C^β	1.46201	1.25025	10.8146	3.22751
* D	C^γ	1.3105	1.285	11.0274	3.27626
* D	$O^{\delta1,\delta2}$	1.23955	1.30143	10.6261	3.23178
E	N	1.98557	1.15871	10.8956	3.64001
E	O	1.85071	1.17845	10.8713	3.46577
E	C	1.9308	1.16974	11.357	3.57748
E	C^α	1.77466	1.19266	11.4089	3.64965
* E	C^β	1.48677	1.24168	11.0907	3.54274
* E	C^γ	1.31626	1.27515	10.8993	3.57997
* E	C^δ	1.7199	1.2982	10.7479	3.88045
* E	$O^{\epsilon1,\epsilon2}$	1.098	1.31389	10.4551	3.89444
F	N	2.26286	1.07153	10.3102	1.80383
F	O	2.23484	1.06709	9.84234	1.93161
F	C	2.20673	1.07025	10.435	1.87167
F	C^α	2.33045	1.04534	10.1424	1.84731
· F	C^β	2.70951	0.994458	9.58106	1.94113
* F	C^γ	3.21173	0.938349	8.82322	2.0412
* F	$C^{\delta1,\delta2}$	3.43458	0.912336	8.25185	2.07026
* F	$C^{\epsilon1,\epsilon2}$	3.97823	0.861718	7.50868	2.15559
* F	C^ζ	4.07383	0.850231	7.34663	2.14493
G	N	1.41659	1.25703	10.7643	1.83934
G	O	1.52184	1.21896	8.89533	1.944456
G	C	1.51989	1.22519	9.73177	1.95465
G	C^α	1.36597	1.26867	9.86165	1.8015
H	N	1.79522	1.14741	10.5185	1.77569
H	O	1.64444	1.17279	10.3303	1.60987
H	C	1.71863	1.1582	10.4955	1.67697
H	C^α	1.74533	1.15543	10.1783	1.73945
· H	C^β	1.79077	1.14146	8.99532	1.79405
· H	C^γ	1.66554	1.16869	8.73623	1.75192
* H	$N^{\delta1}$	1.55861	1.19585	8.51195	1.72934
* H	$C^{\delta2}$	1.56118	1.1889	8.20466	1.66551
* H	$C^{\epsilon1}$	1.38993	1.24323	8.33837	1.613
* H	$N^{\epsilon2}$	1.3804	1.24305	8.17855	1.56715
I	N	2.28888	1.0443	10.0481	1.76938
I	O	1.97195	1.09031	10.7303	1.58805
I	C	2.0243	1.07838	10.7318	1.58331
I	C^α	2.45109	1.01476	9.4227	1.7797
· I	C^β	3.0675	0.940875	8.31378	1.96042
* I	$C^{\gamma1}$	3.95679	0.875253	7.82796	2.22682
* I	$C^{\gamma2}$	3.27028	0.908294	7.47578	1.97036
* I	$C^{\delta1}$	4.95071	0.818597	7.43178	2.43311
K	N	2.73216	1.07917	10.8264	4.74762
K	O	2.10346	1.13886	10.4785	3.95028
K	C	2.33073	1.11818	11.0779	4.28649
K	C^α	2.39826	1.10889	11.0951	4.81372
* K	C^β	2.08623	1.13076	10.6215	5.00503
* K	C^γ	1.72442	1.17117	10.461	4.91766
* K	C^δ	1.41126	1.20814	10.3063	5.11234
* K	C^ϵ	1.15114	1.25083	10.2292	5.15416
* K	N^ζ	0.95605	1.29372	10.1785	5.12301
L	N	2.08193	1.09252	10.6923	1.84865
L	O	2.00599	1.10513	10.0332	1.85942
L	C	2.04411	1.09916	10.5414	1.84819
L	C^α	2.21541	1.06739	10.1249	1.87492
* L	C^β	2.56815	1.01039	8.90099	1.96028
* L	C^γ	3.08358	0.94331	7.82283	2.07451
* L	$C^{\delta1,\delta2}$	3.7841	0.877396	7.04436	2.26302
M	N	2.16412	1.07156	9.2319	1.88134
M	O	2.2221	1.06911	9.223	1.98419
M	C	2.19126	1.07248	9.55179	1.92684
M	C^α	2.3475	1.04142	8.86534	1.93347
· M	C^β	2.57036	0.998159	7.92453	1.93913
· M	C^γ	3.31598	0.903553	6.74281	2.13118
* M	S^δ	4.4474	0.80527	6.1702	2.29823
* M	C^ϵ	4.75168	0.773953	5.79667	2.34705
N	N	2.02814	1.14674	10.2247	3.1657
N	O	1.97925	1.14291	9.10539	3.08941
N	C	2.0731	1.13614	9.72506	3.21494
N	C^α	1.91178	1.16099	9.83299	3.18936
* N	C^β	1.60765	1.2129	9.53132	2.97622
* N	C^γ	1.39266	1.26559	9.86253	2.8308
* N	$O^{\delta1}$	1.3314	1.2813	9.66778	2.75331
* N	$N^{\delta2}$	1.28146	1.29503	9.68075	2.76466

Continued on the next page.

Table S2. – *Continued.*

τ=(Aa,	Atom)	A_τ	μ_τ	β_τ	γ_τ
P	N	1.74989	1.19768	11.1837	3.07058
P	O	1.73507	1.19726	10.6971	3.09794
P	C	1.72868	1.20225	11.3192	3.077
P	C^{α}	1.65503	1.21496	11.1319	2.9767
* P	C^{β}	1.39257	1.26921	10.0829	2.65189
* P	C^{δ}	1.52256	1.23685	10.1116	2.77894
* P	C^{γ}	1.34911	1.27895	9.66413	2.56784
Q	N	2.08951	1.14403	10.8973	3.28799
Q	O	1.91142	1.16703	10.6304	3.07799
Q	C	1.96307	1.16312	11.2172	3.12275
Q	C^{α}	1.90483	1.17245	11.1804	3.2515
* Q	C^{β}	1.66129	1.21203	10.922	3.14764
* Q	C^{δ}	1.34825	1.27416	10.5634	3.22498
* Q	C^{γ}	1.51229	1.23811	10.5453	3.20969
* Q	$O^{\epsilon 1}$	1.30044	1.28201	10.0559	3.22444
* Q	$N^{\epsilon 2}$	1.23968	1.30301	10.4475	3.13179
R	N	2.61297	1.08558	10.7821	3.58399
R	O	2.2283	1.1188	10.1269	3.20002
R	C	2.37103	1.10773	10.6765	3.35447
R	C^{α}	2.40342	1.10845	10.9546	3.5691
* R	C^{β}	2.14991	1.13602	10.7208	3.54819
* R	C^{γ}	2.03587	1.14804	10.4279	3.67619
* R	C^{δ}	1.75977	1.18286	9.88739	3.62322
* R	N^{ϵ}	1.72646	1.18439	9.83642	3.84888
* R	C^{ζ}	1.63791	1.19295	9.59623	3.99212
* R	$N^{\eta 1,\eta 2}$	1.54164	1.20664	9.31346	3.94206
S	N	1.65424	1.19857	9.83601	2.19989
S	O	1.64665	1.20153	9.61894	2.30612
S	C	1.66862	1.19903	9.96855	2.31836
S	C^{α}	1.53058	1.22762	9.73145	2.14097
* S	C^{β}	1.3328	1.28044	9.26341	1.94463
* S	O^{γ}	1.33149	1.28084	9.09863	2.01394
T	N	1.91911	1.1521	10.5342	2.36853
T	O	1.87189	1.15123	9.33962	2.308
T	C	1.90488	1.15021	9.89948	2.34845
T	C^{α}	1.78745	1.17279	10.157	2.26954
* T	C^{β}	1.56428	1.21837	9.83832	2.10378
* T	$O^{\gamma 1}$	1.53926	1.22632	9.78322	2.1719
* T	$C^{\gamma 2}$	1.40288	1.25504	9.2939	1.85524
V	N	2.14248	1.06128	9.61542	1.69647
V	O	1.95298	1.09021	9.79511	1.58572
V	C	2.01885	1.07492	9.627664	1.58672
V	C^{α}	2.31811	1.02622	8.74914	1.72555
· V	C^{β}	2.82571	0.959132	7.78537	1.91297
* V	$C^{\gamma 1,\gamma 2}$	3.4067	0.898944	7.10407	2.09198
W	N	2.16666	1.06852	9.68157	1.8162
W	O	2.52022	1.02418	8.45761	2.10325
W	C	2.60672	1.01789	8.88978	2.12404
W	C^{α}	2.48678	1.02519	9.18021	1.96748
· W	C^{β}	2.74637	0.993851	9.08366	2.03041
· W	C^{γ}	2.87076	0.992432	9.78947	2.12948
· W	$C^{\delta 1}$	2.39333	1.04045	9.45923	1.95456
* W	$C^{\delta 2}$	3.2157	0.97072	10.5317	2.25817
· W	$N^{\epsilon 1}$	2.47356	1.03365	9.33899	2.03229
· W	$C^{\epsilon 2}$	2.9114	0.991737	9.76106	2.18207
* W	$C^{\epsilon 3}$	3.66434	0.93326	10.0902	2.33315
· W	$C^{\zeta 2}$	2.87099	0.988483	9.15555	2.14396
* W	$C^{\zeta 3}$	3.60627	0.928426	9.44348	2.28163
· W	$C^{\eta 2}$	3.14229	0.959377	9.06995	2.16779
Y	N	2.45871	1.06558	10.5233	2.36149
Y	O	2.55032	1.0485	9.64581	2.38468
Y	C	2.57736	1.05001	10.2519	2.39466
Y	C^{α}	2.68635	1.0426	10.4916	2.47518
· Y	C^{β}	3.1316	1.00069	9.91091	2.65577
· Y	C^{γ}	2.96367	1.01834	10.1356	2.64251
· Y	$C^{\delta 1,\delta 2}$	2.72005	1.0364	9.81383	2.52674
* Y	$C^{\epsilon 1,\epsilon 2}$	2.35074	1.07434	9.54375	2.36893
* Y	C^{ζ}	2.20694	1.09349	9.5077	2.31764
* Y	O^{η}	1.8242	1.1511	9.06886	2.09818
