# Supplementary material for: Prediction of functionally important residues in globular proteins from unusual central distances of amino acids
Source: BMC Struct Biol. 2011 Sep 18;11:34. doi: 10.1186/1472-6807-11-34 (PMC3188475; doi:10.1186/1472-6807-11-34)
Supplement: Additional file 5 — Probability densities of Cα and distal side chain atoms of 20 amino acids. [file 1472-6807-11-34-S5.PDF]

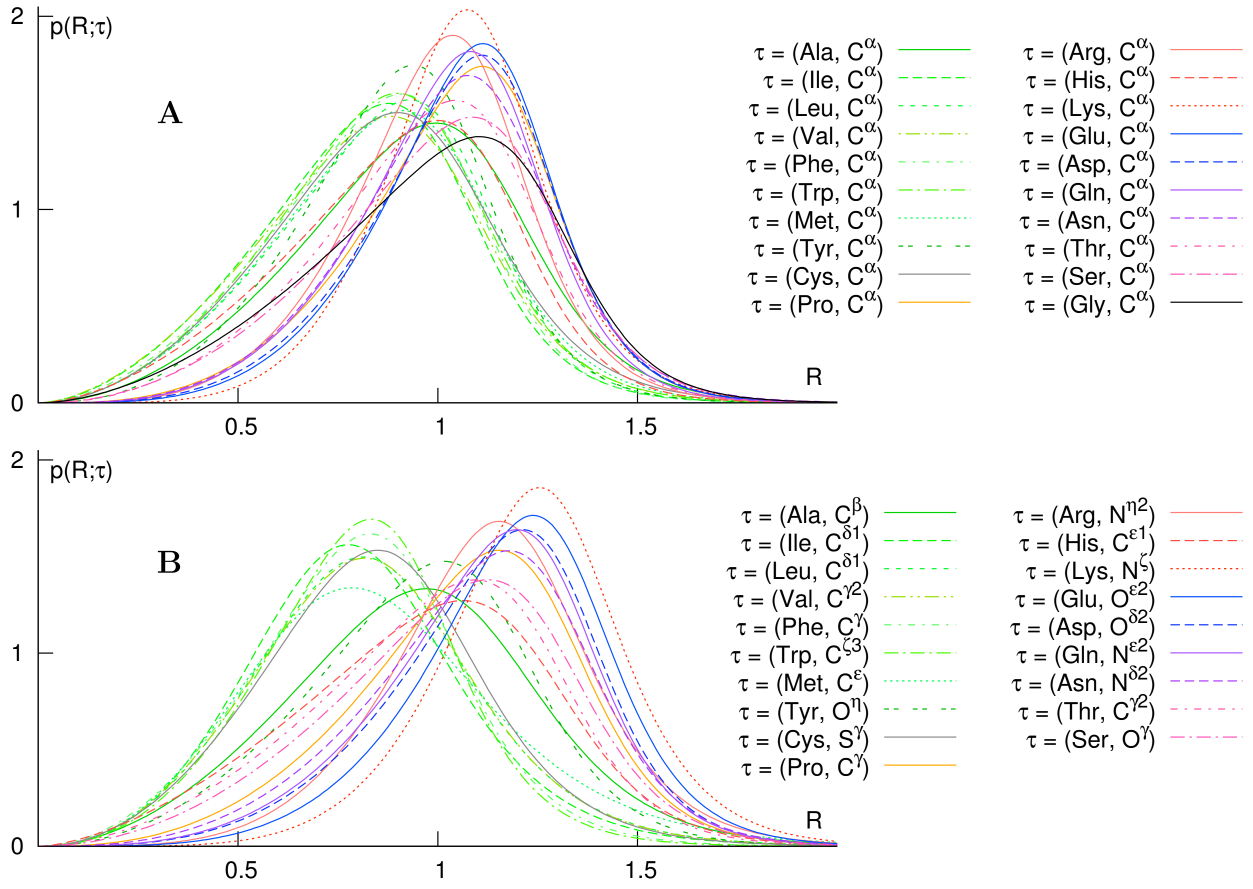

Figure S3. Distributions of central distances of Cα (**A**) and distal side chain atoms (**B**) of all amino acids. Curves for amino acids with hydrophobic side chains are green, polar charged – red and blue, polar uncharged – pink and violet.
