# Supplementary material for: Prediction of functionally important residues in globular proteins from unusual central distances of amino acids
Source: BMC Struct Biol. 2011 Sep 18;11:34. doi: 10.1186/1472-6807-11-34 (PMC3188475; doi:10.1186/1472-6807-11-34)
Supplement: Additional file 6 — Probability densities of Cα and distal side chain atoms of Cys. [file 1472-6807-11-34-S6.PDF]

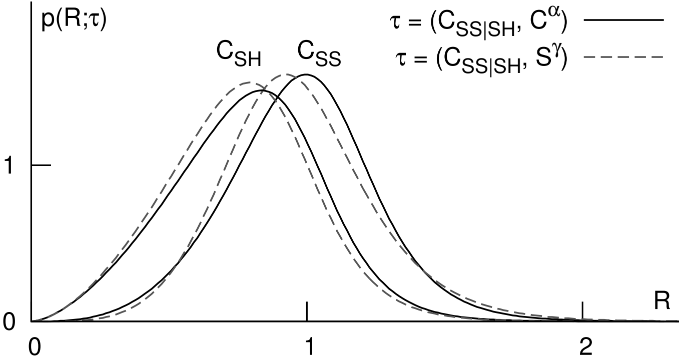

Figure S4. Probability densities of C^{α} and distal side chain atoms of Cys. Two cases are shown separately: Cys bridged (C_{SS}) and not bridged (C_{SH}) by disulfide bonds.
