# Supplementary material for: Prediction of functionally important residues in globular proteins from unusual central distances of amino acids
Source: BMC Struct Biol. 2011 Sep 18;11:34. doi: 10.1186/1472-6807-11-34 (PMC3188475; doi:10.1186/1472-6807-11-34)
Supplement: Additional file 7 — Details on the efficiency of the SurpResi applied to the LB48 test set. [file 1472-6807-11-34-S7.PDF]

Table S3. Geometrical and functional characteristics of structures from the non-redundant apo-holo set created by Hwang and Schroeder (*BMC Struct Biol* 6:19 (2006)) and performance of two binding site recognition methods for two cutoff distances. Geometrical descriptors, aspericity (Asph.) and compactness (Comp.), are reported for apoproteins. The list is ordered according to the increasing asphericity. When a method was unable to find a site, the rank is ∞. Enzyme class assignments according to the Catalytic Site Atlas (*Nucleic Acids Res* 32:D129-33 (2004)) version 2.2.12 (January 2010).

PDB and chain ID			Enzyme class or the non-substrate ligand	Geometry		FOD		SurpResi	
Apo	Holo —	Ligands		Asph.	Comp.	4 Å	6 Å	4 Å	6 Å
1nna A	1ivd A	{FUL, ST1, NAG, MAN	3.2.1.18	0.0144	0.5617	1	1	1	1
2sil A	2sim A	DAN	3.2.1.18	0.0198	0.555	1	2	1	1
2ctb A	2ctc A	HFA	3.4.17.1	0.0236	0.5836	∞	2	1	1
5cpa A	7cpa A	FVF	3.4.17.1	0.0237	0.5744	∞	1	∞	1
1brq A	1rbp A	RTL	retinol	0.0237	0.5431	1	1	∞	2
1hxf H	1dwd H	MID	3.4.21.5	0.0241	0.536	∞	1	2	2
2cba A	2h4n A	AZM	4.2.1.1	0.025	0.5497	1	1	1	1
4ca2 A	1okm A	SAB	4.2.1.1	0.0251	0.5592	1	1	∞	1
1cge A	1hfc A	PLH	3.4.24.7	0.037	0.5682	1	1	1	1
1esa A	1inc A	ICL	3.4.21.36	0.0375	0.5595	∞	1	∞	1
1stn A	1snc A	THP	3.1.31.1	0.0378	0.56	1	1	1	1
1chg A	3gch C	OAC	3.4.21.1	0.0397	0.5645	∞	∞	∞	2
1ypi A	2ypi A	PGA	5.3.1.1	0.041	0.5401	1	1	1	1
3tms A	1bid A	FMT,UMP	2.1.1.45	0.0428	0.52	1	1	1	1
1ifb A	2ifb A	PLM	fatty acid	0.0446	0.6057	1	1	1	1
1qif A	1acj A	THA	3.1.1.7	0.0451	0.4984	∞	2	1	1
1ula A	1ulb A	GUN	2.4.2.1	0.0562	0.5017	∞	∞	∞	1
1ime A	1imb A	LIP	3.1.3.25	0.0588	0.5353	1	1	1	1
3ptn A	3ptb A	BEN	3.4.21.4	0.0602	0.598	1	1	1	1
2tga A	1mtw A	DX9	3.4.21.4	0.0617	0.6043	∞	∞	∞	2
1bya A	1byb A	GLC	3.2.1.2	0.0646	0.51	1	1	1	1
1krn A	2pk4 A	ACA	3.4.21.7	0.0764	0.6402	∞	∞	1	1
1phc A	1phd A	HEM,PIM	1.14.15.1	0.0795	0.508	1	1	4	4
5dfr A	4dfr A	MTX	1.5.1.3	0.0806	0.5479	1	1	1	1
1djb A	1blh A	FOS	3.5.2.6	0.0833	0.5586	1	1	2	1
2fbp B	1fbp B	AMP,F6P	3.1.3.11	0.0864	0.4873	1	2	1	1
1ahc A	1mrg A	ADN	3.2.2.22	0.0937	0.5505	∞	1	3	1
1pdy A	1pdz A	ACE,PGA	4.2.1.11	0.096	0.5315	2	1	2	1
2ctv A	5cna A	MMA	saccharide	0.0979	0.5612	∞	∞	∞	∞
1hsi A	1ida A	{QND, PR0, PY2, PPL	{3.4.23.47, 2.7.7.7, 3.1.26.13, 2.7.7.49	0.1121	0.5362	1	1	2	1
1bbs A	1rne A	NGA,C60	3.4.23.15	0.1159	0.5135	1	1	1	1
1hel A	1hew A	NAG	3.2.1.17	0.1204	0.6043	∞	1	5	5
3phv A	4phv A	VAC	{2.7.7.49, 3.4.23.16, 2.7.7.7, 3.1.26.13	0.1219	0.5386	∞	∞	∞	2
6ins E	3mth A	MPB	benzoic acid ester	0.1259	0.5948	∞	∞	∞	∞
3app A	1apu E	IVA,STA,EHN	3.4.23.20	0.1274	0.5507	1	1	1	1
3lck A	1qpe A	PP2,PTR	2.7.10.2	0.1356	0.4987	∞	1	∞	2
1psn A	1pso E	IVA,STA	3.4.23.1	0.1417	0.5352	2	2	2	1
8rat A	1rob A	C2P	3.1.27.5	0.1427	0.5795	1	1	2	1
1a6u H	1a6w H	NIP	iodonitrophenylacetyl-aminocaproic acid	0.1446	0.6371	∞	∞	∞	2
1swb A	1stp A	BTN	biotin	0.145	0.542	1	1	∞	∞
1pts A	1srf A	MTB	azobenzoic acid	0.1553	0.5564	1	1	1	1
3p2p A	5p2p A	DHG	3.1.1.4	0.1667	0.5674	1	1	1	1
2rta A	1stp A	BTN	biotin	0.1703	0.5332	∞	1	∞	∞
8adh A	1cdo A	NAD	1.1.1.1	0.1795	0.5026	1	1	1	1
1l3f E	2tmn E	0FA	3.4.24.27	0.1966	0.573	∞	1	1	1
1npc A	1hyt A	DMS,BZS	3.4.24.28	0.2032	0.557	1	1	1	1
1gcg A	1gca A	GAL	aldohexose	0.2881	0.5325	1	1	4	4
1a4j B	1igj D	DGX	digoxin	0.4645	0.4737	∞	∞	∞	∞
